# Inhibition of KMO Ameliorates Myocardial Ischemia Injury via Maintaining Mitochondrial Fusion and Fission Balance

**DOI:** 10.7150/ijbs.83392

**Published:** 2023-06-12

**Authors:** Qiong Lai, Lingling Wu, Shuhong Dong, Xiaozhou Zhu, Zhaoyang Fan, Junping Kou, Fuming Liu, Boyang Yu, Fang Li

**Affiliations:** 1 Jiangsu Key Laboratory of TCM Evaluation and Translational Research, Research Center for Traceability and Standardization of TCMs, School of Traditional Chinese Pharmacy, China Pharmaceutical University, 639 Longmian Road, Nanjing, 211198, China.; 2 Jiangsu Province Hospital of Chinese Medicine, Affiliated Hospital of Nanjing University of Chinese Medicine, Nanjing, 210029, China.

**Keywords:** Myocardial ischemia, Xanthurenic acid, Kynurenine 3-monooxygenase, Mitochondrial fusion and fission, Ginsenoside Rb3

## Abstract

Looking for early diagnostic markers and therapeutic targets is the key to ensuring prompt treatment of myocardial ischemia (MI). Here, a novel biomarker xanthurenic acid (XA) was identified based on metabolomics and exhibited high sensitivity and specificity in the diagnosis of MI patients. Additionally, the elevation of XA was proved to induce myocardial injury *in vivo* by promoting myocardial apoptosis and ferroptosis. Combining metabolomics and transcriptional data further revealed that kynurenine 3-monooxygenase (KMO) profoundly increased in MI mice, and was closely associated with the elevation of XA. More importantly, pharmacological or heart-specific inhibition of KMO obviously suppressed the elevation of XA and profoundly ameliorated the OGD-induced cardiomyocytes injury and the ligation-induced MI injury. Mechanistically, KMO inhibition effectively restrained myocardial apoptosis and ferroptosis by modulating mitochondrial fission and fusion. In addition, virtual screening and experimental validation were adopted to identify ginsenoside Rb3 as a novel inhibitor of KMO and exhibited great cardioprotective effects by regulating mitochondrial dynamical balance. Taken together, targeting KMO may provide a new approach for the clinical treatment of MI through maintaining mitochondrial fusion and fission balance, and ginsenoside Rb3 showed great potential to be developed as a novel therapeutic drug targeting KMO.

## Introduction

Cardiovascular disease (CVD) is responsible for a staggering ~25 to 30% of mortality worldwide 1, and it is estimated that the number of world people dying from myocardial infarction is about 23.6 million, which may produce immense health and economic burdens in the world 2. Myocardial ischemia (MI) is an initial and prominent factor of myocardial infarction, caused by a decrease in myocardial oxygen supply and/or an increase in myocardial oxygen demand 3-5. Timely treatment of myocardial ischemia can reduce rates of myocardial injury and prevent it from progressing into myocardial infarction, heart failure, and some other cardiovascular diseases 6, 7. However, roughly 75% of ischemic episodes have few symptoms or signs (i.e., subclinical ischemia), and the heart always suffers irreversibly impairment without conscious awareness 8-10. When the myocardium suffers ischemic damage, it can only be targeted by timely reperfusion therapy in clinics. Unfortunately, the paradoxical myocardial reperfusion injury may extend the final infarct size 11, and it requires adjunctive treatment strategies beyond reperfusion to improve clinical outcomes. Therefore, looking for more accurate early diagnostic markers, effective potential therapeutic targets, and drugs are essential to ensure prompt medical attention to myocardial ischemia.

Metabolomics technology provides a way to offer insight into the disease progression through discovering potential metabolic markers and elucidating the undiscovered relevant metabolic pathways 12-14. There are numerous studies that have proved the successful application of metabolomics in discovering biomarkers for cardiovascular diseases 15-18. Hence, metabolomics is a good way to excavate the diagnostic biomarkers for myocardial ischemia. In addition, this might be a good attempt to combine metabolomics with transcriptomics for screening new targets for the treatment of myocardial ischemia as well.

In this study, experimental animal mice and clinical participants were performed to identify the potential metabolic biomarkers of myocardial ischemia using a combination of untargeted and targeted metabolomics analysis. The present study found that the level of metabolite xanthurenic acid (XA) was significantly elevated in the early stage of myocardial ischemia in mice and patients. More importantly, xanthurenic acid showed great potential in the diagnosis of myocardial ischemia patients. Subsequently, a functional metabolomics strategy was adopted to reveal the potential function of xanthurenic acid, as well as discover its related metabolic enzyme. Our results demonstrated that inhibition of kynurenine 3-monooxygenase (KMO) profoundly ameliorated myocardial injury by maintaining mitochondrial fusion and fission balance, which may reveal a novel potential therapeutic strategy for myocardial ischemia. Besides, ginsenoside Rb3 was screened as the novel inhibitor of KMO, and it also exhibited an excellent therapeutic effect on myocardial ischemia by maintaining mitochondrial fusion and fission balance.

## Material and methods

### Reagent

Ginsenoside Rb3 with high purity (> 98 %) was purchased from ChemFaces Biochemical Co., Ltd. (Wuhan, China). Ultrapure water was obtained from a Milli-Q purification system (Milford, MA, USA). Methyl thiazolyl tetrazolium (MTT) was purchased from Amersco (Washington, USA). N-acetyl-L-cysteine (NAC) was obtained from Sigma-Aldrich (St. Louis, MO, USA). Lactate dehydrogenase (LDH) assay kits (A020-2-2) were obtained from Nanjing Jiancheng Bioengineering Institute (Nanjing, China). Brain natriuretic peptide (BNP) kit (JEB-12888), high-sensitivity C-reactive protein (hs-CRP) kit (JEB-12578), tumor necrosis factor-α (TNF-α) kit (JEB-12474), malondialdehyde (MDA) kit (JEB-12601), and nitric oxide (NO) assay kit (JEB-12547) were obtained from Nanjing Jin Yibai Biological Technology Co. Ltd.

### Animals and MI model

Eight- to 10-week-old male C57BL/6 (wild-type) mice were obtained from the Model Animal Research Centre of Yangzhou University and kept in SPF level animal lab. All procedures and protocols were administrated according to the National Institutes of Health Guidelines for the Care and Use of Laboratory Animals and approved by the Animal Ethics Committee of China Pharmaceutical University (Nanjing, China) (Approval No. SYXK2021-0010). All the animal studies were designed to produce equally sized groups using randomized and blinded analysis. Mice were killed using CO_2_ at the end of the experiments. The myocardial ischemia model was induced by left anterior descending coronary artery ligation (CAL) 19. Briefly, mice were anesthetized with a single intraperitoneal injection of ketamine (60 mg/kg) and xylazine hydrochloride (100 mg/kg). Then the mice were connected to the artificial ventilator until the operation is completed within 3 min. The heart was exposed via a left thoracotomy, and a slipknot was tied around the left anterior descending coronary artery 3-4 mm from its origin utilizing a 6-0 silk suture. Sham-operated mice were administrated with the same surgical procedures without ligating the left anterior descending coronary artery. ST-segment elevation was used to demonstrate the success of the myocardial ischemia through an electrocardiogram monitor. In the drug administration group, the drug was dissolved in normal saline and administered by gavage or intraperitoneal injection according to the experimental measurement. Serum, plasma, and urine samples were obtained after overnight fasting and stored at -80 ºC for metabolite analyses. And all organs were washed with normal saline after removal and immediately stored at -80 ºC. The experimental groups of this study as shown in **Supplementary Figure S1** (n = 7-13 / group).

### Human samples

A total of 64 patients with myocardial ischemia and normal persons were enrolled from Jiangsu Province Hospital of Chinese Medicine (normal persons: 23, myocardial ischemia patients: 41). Patients with liver disease, active vasculitis, gastrointestinal pathology, cancer, or other diseases were excluded from the study. Informed consent was acquired from all participants. This study was approved by the Ethical Committee (No. 2019NL-089-02). The specific patient characteristics are represented in **Supplementary Table S1**. Urine and serum samples were obtained after overnight fasting and stored at -80 ºC for metabolite analyses.

### Echocardiographic measurement

An echocardiographic system consisting of a Vevo 2100 Imaging System (Visual Sonics, Toronto, Canada) equipped with a 30 MHz transducer was used to assess the cardiac function of experimental mice. The mice were transferred to dorsal recumbency and placed on the imaging platform when the mice were fully anesthetized by 2.5% isoflurane in O_2_ gas 20. Indicators of cardiac function measured in this study are as follows: Left ventricular (LV) ejection fraction (EF), LV fractional shortening (FS), stroke volume (SV), LV septum thickness; diastole (IVS;d), LV diameter; diastole (LVID;d), LV mass (corrected).

### Histopathologic examination

The tissues were fixed in 4% paraformaldehyde solution, embedded in paraffin, sliced into pieces 5 μm thick, and stained with hematoxylin and eosin (H&E) and Masson's trichrome staining. To detect collagen fibers, the tissue sections embedded in paraffin (2-3 mm) were stained in 0.1% Sirius-red F3B in saturated picric acid. The histopathological changes were detected by an optical microscope 21. All histopathologic evaluation was performed by two pathologists in a double-blinded fashion.

### Immunohistochemistry

After fixed in the 4% paraformaldehyde and embedded in paraffin, the tissues were sectioned at 4 µm thicknesses, deparaffinized and rehydrated in PBS, and incubated with 3% hydrogen peroxide to block endogenous peroxidase activity. And then, the sections were incubated for 1 h at 37 °C with blocking liquid (Beyotime, Shanghai, China). The primary antibodies were dropwise added to the sections and kept at 4 °C for 24 h. After washing, sections were incubated with the HRP-conjugated secondary antibody (1:200, Biogot Technology, Nanjing, China) at 37 °C for 1 h. After incubated with DAB, counterstained with hematoxylin, a light microscope (DX45, Olympus Microsystems Ltd., Japanese) was applied to observe the dehydrated sections and finally imaged at 400X magnification 22.

### Untargeted Metabolomics Analysis

#### HPLC-Q-TOF MS analysis

Serum, plasma, and urine were collected and immediately frozen at -80 ºC until analyses. Metabolite separation was conducted using Agilent Technologies 6530 Accurate-Mass Q-TOF LC/MS (USA). The mobile phases are water + 0.1% formic acid (eluent A) and acetonitrile + 0.1% formic acid (eluent B). TSK-GEL Amide-80 column (150×2.0 mm i.d., 5 μm) was adopted to analyze biological samples. The specific methods of sample pretreatment and the conditions of liquid phase analysis referred to our previous research 23.

#### Method assessment

The robustness and repeatability of the HPLC-Q-TOF MS analysis were examined by preparing mixed quality control samples (QC) 24. QC test samples were pre-treated with reference to the test samples and randomly injected throughout the test sequence list. The unsupervised PCA analysis **(Supplementary Figure S2A)** of QC samples and the overlap of total ion chromatograms (TICs) **(Supplementary Figure S2B)** verified that the current analytical methods have good repeatability and robustness. Meanwhile, the RSD% values of retention time and peak area of five different ions extracted from TICs of QC sample were statistically lower than 4% and 19% respectively, which further verified the good reproducibility of the experimental method and instrument stability **(Supplementary Table S2)**. In addition, the representative total ion chromatograms (TICs) of plasma, urine, and serum samples of each group were shown in **Supplementary Figure S2C**.

#### Data processing

The raw data obtained from the Q-TOF LC/MS ESI^+^ analysis was subjected to data preprocessing using R software, and the data were further screened according to the 80% principle. The processed data were imported into SIMCA-P 14.1 (Umetrics, Sweden) for orthogonal partial least-squares discriminant analysis (OPLS-DA) and PCA analysis. The metabolites with significant differences were screened by *p*-value < 0.05 and variables importance in the projection (VIP) value > 1.0.

#### Metabolite identification

The structural information of the screened differential metabolite ions was matched using the online databases HMDB, METLIN, and MassBank.

### Targeted analysis by LC-MS/MS

#### Pretreatment of mice and clinical samples

50 μL of urine, serum, and six kinds of organ tissue grinds were taken, added 140 μL of methanol and 10 μL of internal standard solution (p-aminosalicylic acid) for protein precipitation. After high-speed centrifugation, the supernate was taken for further analysis.

#### LC-MS/MS analysis

The analysis was carried out on a triple quadrupole LC-MS/MS system. The mobile phases were consistent with untargeted metabolomics analysis. Chromatographic separation of all test samples was conducted on a TSK-GEL Amide-80 column (150×2.0 mm i.d., 5 μm). Therein, the gradient program of 2-arachidonoylglycerophosphocholine in serum starts with 80-75% B at 0-3 min, 75-60% B at 3-8 min, 60-50% B at 8-10 min, 50% B at 10-12 min, and then back to initial conditions, with 5 min for equilibration. Besides, the gradient program of fructoselysine, 7-methylguanine, and xanthurenic acid in urine and six kinds of organ tissues start with 70-65% B at 0-3 min, 65-60% B at 3-8 min, 60-50% B at 8-10 min, 50% B at 10-22 min, and then back to initial conditions, with 5 min for equilibration. The flow rate was 0.2 mL/min and the injection volume was 5 μL.

All metabolites were identified in the multiple-reaction-monitoring (MRM) of positive ion mode (ESI+), and metabolite identification and quantification were performed with standard substance of fructoselysine, 7-methylguanine, xanthurenic acid, and 2-arachidonoylglycerophosphocholine. Masshunter software was used for data acquisition and processing. The target LC-MS/MS method has been verified in linearity, precision, accuracy, extraction recovery, matrix effects, and mass spectrometry parameters, as shown in **Supplementary Table S3-4**. The precision and accuracy were below 15% and the linear curve fitted well, which reflected the high reliability and accuracy of these methods **(Supplementary Figure S3)**.

### RNA-seq and genome-wide transcriptome analysis

Total RNA was extracted from heart tissues using TRIzol (Invitrogen) and then purified. The raw data was handled through an in-house Perl scripts and the reads including the adapter are removed to obtain clean data. And then, STAR (v2.5.1b) was used to establish the index of the reference genome, align the pairs of clean reads to the reference genome, and htseqv0 6.0 was used to count the readings corresponding to each gene. EdgeR R package (3.12.1) was used to analyze and screen differentially expressed genes, and clusterProfiler R package was used in GO and KEGG pathway analysis 25.

### Construction and infection of recombinant AAV-KMO

Serotype 9 adeno-associated virus (pAAV9) vectors carrying a specific sequence inhibiting KMO (PGMAAV-10261) and negative control (NC) (PGMAAV-10261) were constructed by Genomeditech Co. (Shanghai, China). Control virus particles and pAAV9-KMO (×10^11^ pfu/mL) were injected directly into the left ventricular free wall of mice using a 30-gauge needle. At the third week after injection, after verifying the successful genetic transfection of AAV *in vivo* using immunohistochemical methods, we performed sham and CAL surgery on experimental mice and sacrificed them two weeks later.

### Immunofluorescence staining

The frozen sections of animal tissues were taken out and placed at room temperature, washed with phosphate buffered saline, and fixed with 4% paraformaldehyde, and then the tissue sections were permeabilized and sealed. Mfn2 (1: 200, Santa Cruz, #sc-515647), OPA1 (1:200, Proteintech, #27733-1-AP), p-Drp1 (1: 200, Cell Signaling Technology, #3455), Pink1 (1:200, Abcam, #ab216144) and Parkin (1:200, Abcam, #ab15494) antibodies were added to incubate overnight at 4 ºC and fluorescent secondary antibodies were added to incubate for 2 hours. DAPI (Beyotime Biotechnology, Shanghai, China) was used to dye the nucleus, and finally, a confocal laser scanning microscope (LSM700, Zeiss, Jena, Germany) was used for fluorescence observation and photography. In addition, the cells were washed with ice-cold PBS, and the intracellular mitochondria were localized with the MitoTracker® Deep Red. The subsequent procedure of cell immunofluorescence staining is consistent with animal tissues.

The mitochondrial structure in the image was separated and processed. Image J was used to calculate the mitochondrial length, aspect ratio (AR), and form factor (FF) values. AR describes changes in mitochondrial length, while FF describes changes in length and degree of branching. Average mitochondrial length, AR, and FF values were calculated from five random images for each experiment, with smaller values representing increased mitochondrial debris 26.

### Quantification of mitochondrial DNA (mtDNA) content

The total DNA of H9c2 cells was extracted by using FastPure®Cell/Tissue DNA Isolation Mini Kit (DC102, Vazyme, Nanjing, China). mtDNA content was measured by real-time fluorescence quantitative PCR. Real-time PCR was performed on the Mastercycler EP Realplex PCR system (Bio-Rad) and the relative mtDNA expression levels were normalized to that of β-globin using the 2^-ΔΔCT^ method. The primers used in this study were shown below: mtDNA (Sense: 5'-GAUUACAGUCAGCAAUAUA-3'; Antisense: 5'-GATGGGGCCGGTAGGTCGATAAAGGAG-3'); β-globin (Sense: 5'-CCAATCTGCTCACACAGG-3'; Antisense: 5'-CACCTTTCCCCACAGG-3') 27, 28.

### Western blot analysis

As previously described 29, proteins were extracted from cells and hearts and quantified by a BCA protein assay kit. The same amounts of proteins (35 μg) were separated through 10%-15% SDS-PAGE and transferred to polyvinylidene fluoride (PVDF) membranes (Millipore Corporation, Billerica, Ma, USA) via electroblotting. After blocking, the primary antibody was added and incubated at 4 ℃ overnight: cleave caspase3 (1:2000, Cell Signaling Technology, #9664), FHC (1:1000, Affinity, #DF6278), FLC (1:1000, Abclone, #A21962), GPX4 (1: 1000, Proteintech, #67763-1-Ig), ACSL4 (1:1000, Abclone, #A6826), KMO (1:1000, Proteintech, #10698-1-AP), Mfn2 (1: 1000, Santa Cruz, #sc-515647), OPA1 (1:1000, Proteintech, #27733-1-AP), Phospho-Drp1 (1: 1000, Cell Signaling Technology, #3455), β-actin (1:1000, ZenBio, #250136), Drp1 (1:1000, Abcam, #ab184247), Pink1 (1:1000, Abcam, #ab216144), Parkin (1:1000, Abcam, #ab77924), LC3B (1:1000, Cell Signaling Technology, #43566), P62 (1:1000, Abcam, #ab109012), Beclin 1 (1:1000, Proteintech, #11306-1-AP). And then peroxidase-conjugated secondary antibody (1:10000, #BS13278, #BS12478, Bioworld, Louis Park, MN, USA) was added. Finally, bands were examined by an ECL reagent (Vazyme Biotech, Nanjing, China) and analyzed with Image Lab™ Software (version 4.1, Bio-Rad).

### Transmission electron microscopy

Mouse hearts were cut into small pieces and placed in 2.5% glutaraldehyde for fixation, followed by continued fixation in 0.1 M cacodylate buffer for 1 h. The sections were dehydrated in acetone and embedded in Epon. Subsequently, ultrathin sections were stained with uranyl acetate and lead citrate and observed by LEO 906 (Zeiss) electron microscope operating at 60 kV. And digital electron micrographs were randomly taken at 5000x and 1700x magnification for each group of this study, respectively 30.

### OGD injury model *in vitro*

The rat H9c2 cells (Cell Bank of the Chinese Academy of Sciences, Shanghai, China) were cultured in DMEM (Gibco, Grand Island, NY, USA) including 10% fetal bovine serum (FBS), 100 μg/mL streptomycin, 100 U/mL penicillin. The oxygen and glucose deprived (OGD) method was used to simulate ischemic injury *in vitro*. And the specific operation was to incubate the cells with glucose-free DMEM and culture in an incubator with a hypoxic environment of 37 °C, 94% N_2_, 1% O_2_, and 5% CO_2_. And cell viability of H9c2 cells was determined using 3-(4,5-dime thylthiazol-2-yl)-2,5-diphenyltetrazolium bromide (MTT) method (Beyotime, Nanjing, China).

### siRNA transfection

siRNA targeting specific proteins and control siRNA were transfected into H9c2 cells according to the manufacturer's instructions 31. Briefly, cells were transfected with 20 nM KMO siRNA (Genomeditech, Shanghai, China) diluted with Hyper Transfection Super (ThikarrowTM Scientific Groups, Nanjing, China). 48 h after transfection, cells were collected and the KMO siRNA efficiency was measured by western blotting. The siRNA oligos sequence of KMO was shown below:

Rat-KMO-si-1 (Sense: 5'-GAUUACAGUCAGCAAUAUA-3'; Antisense: 5'-UAUAUUGCUGACUGUAAUC-3'); Rat-KMO-si-2 (Sense: 5'-GCUUCCAACGCAUAGUGAU-3'; Antisense: 5'-AUCACUAUGCGUUGGAAGC-3'); Rat-KMO-si-3 (Sense: 5'-GUCGCCUUCACCAGAAUAA-3'; Antisense: 5'-UUAUUCUGGUGAAGGCGAC-3').

### Molecular Docking

The Autodock Vina was used to investigate the interactions between the compounds and the KMO protein. The protein structure of KMO (PDB ID: 5X68) was obtained from the PDB database (https://www.rcsb.org/) in PDB format. The crystal structure of receptor molecules Ginsenoside Rb3, Astragaloside IV, Salvianic acid C, Ginsenoside Rg3, Cryptotanshinone, and Tanshinone IIA was downloaded from TCMSP (https://tcmsp-e.com/) with mol2 format. Subsequently, the structure was pretreated with open-source Pymol™, including removing the co-crystallized ligand and selecting water molecules and cofactors. The docking process was finished via Autodock Vina. The accurate prediction of active sites was predicted by using the online-database Protein Plus (https://proteins.plus/) 32.

### Surface plasmon resonance (SPR) analysis

The Surface Plasmon Resonance (SPR) method was used in conjunction with Biacore T200 (GE Healthcare, Uppsala, Sweden) to measure the binding affinities at 25 ºC. Recombinant human Kynurenine 3-hydroxylase (KMO) protein (OriGene Technologies Inc., Rockville, MD, USA) was immobilized on the CM5 sensor chip (GE Healthcare, Uppsala, Sweden) by amine-coupling method to achieve the target density of 10000 resonance units (RU). The PBS-P (10 mM sodium phosphate, 150 mM NaCl, 0.005% Tween-20, pH 7.4) was used as a running buffer. Then different concentrations of the compounds (0.1953125 µM, 0.390625 µM, 0.78125 µM, 1.5625 µM, 3.125 µM) were injected at a flow rate of 30 μL/min. Chip platforms were washed with a running buffer and 50% DMSO. The binding affinities were analyzed with the Biacore evaluation software (T200 Version 2.0) by curve fitting using a 1:1 binding model 33.

### Statistical analysis

All data were represented as mean ± SD. GraphPad Prism 8 software (San Diego, CA, USA) was mainly used for data statistical analysis. All data were tested for normal distribution and equal variance. Sample sizes subjected to statistical analysis at least n = 5 per group. Comparisons between two groups were made using Student's two-tailed t-test, and one-way analysis of variance (ANOVA) was used for multiple comparisons (followed by Bonferroni's post hoc or Holm-Sidak's multiple comparisons tests). Differences were considered significant at *p* < 0.05. Besides, receiver operating characteristic (ROC) and area under the curve (AUC) analysis were used to evaluate the sensitivity, specificity, and Youden's index of metabolic markers.

## Results

### Comprehensive metabolites profiles in the serum, plasma, and urine of early stage myocardial ischemia

Coronary artery ligation (CAL) was performed to induce myocardial ischemia in mice. CAL successfully contributed to cardiac dysfunction and subsequently myocardial damage in mice. Cardiac contractile and diastolic function showed a significant decrease **(Supplementary Figure S4A, Supplementary Table S5)**. Simultaneously, morphological damage in heart tissues became increasingly severe after 14-21 days **(Supplementary Figure S4B)**. Thus, the time period of ischemia 14-21 days was adopted in our further research in MI mice.

**Figure 1:**
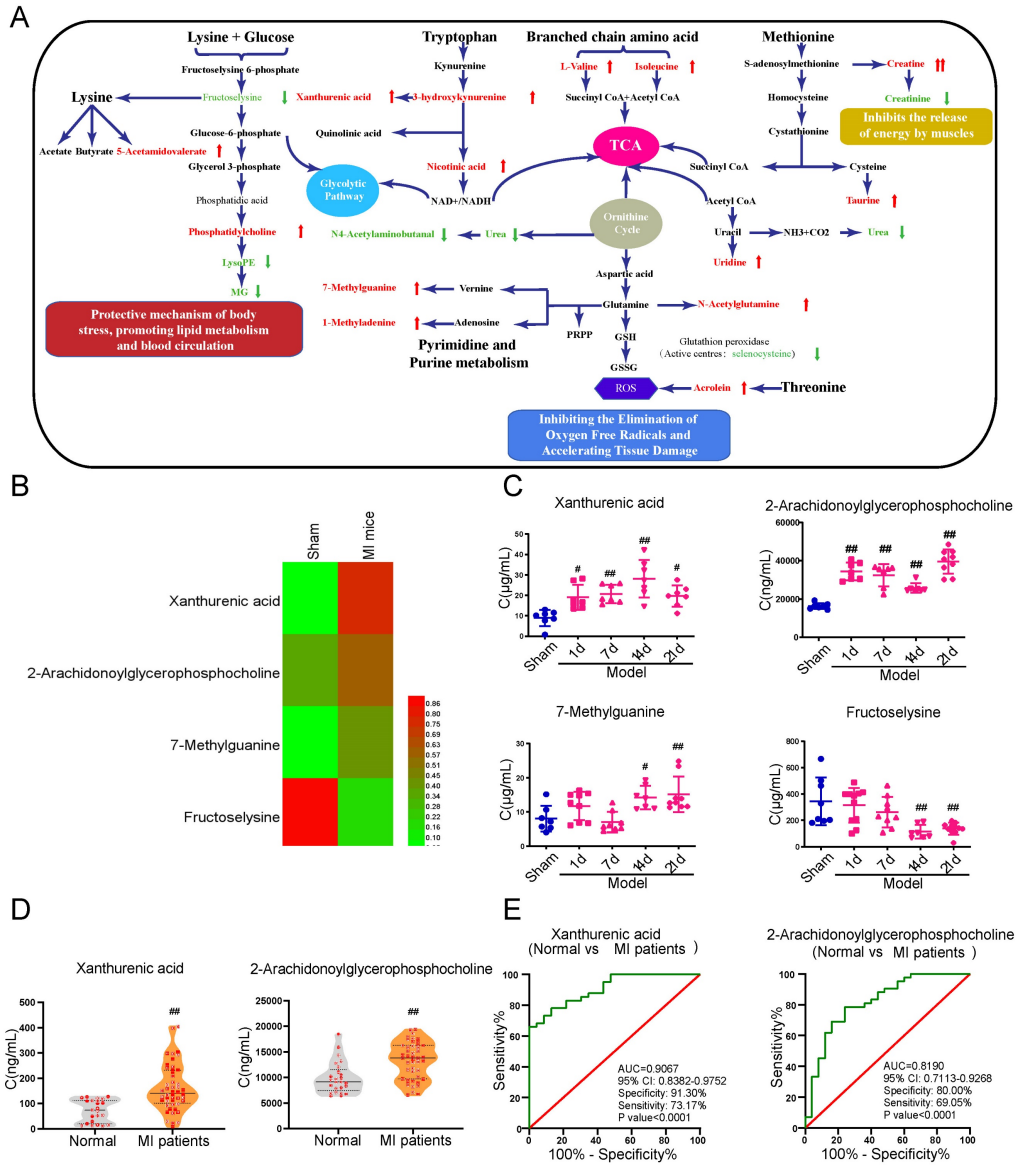
** Xanthurenic acid is identified as the metabolic marker of MI. A.** Metabolite pathway of MI. Compared with the sham group, elevated metabolites in MI mice were labeled red, and the reduced metabolites were labeled green. **B.** Results of heat map analysis of the four potential metabolic biomarkers. Red represents high content and green represents low content (n = 6). **C**. Changes in the content of xanthurenic acid (XA), 2-arachidonoylglycerophosphocholine, 7-methylguanine, and fructoselysine in sham and MI model groups mice during CAL 3 weeks (n = 7-10). **D**. Changes in the content of xanthurenic acid, 2-arachidonoylglycerophosphocholine in normal healthy persons (n = 23) and MI patients (n = 41). All metabolites in biological samples were detected by LC-MS. **E**. Evaluation of sensitivity and specificity of xanthurenic acid and 2-arachidonoylglycerophosphocholine in the diagnosis of MI patients. Analysis of receiver operating characteristic (ROC) curves based on the targeted quantitative results of metabolites in clinical samples. Data are presented as mean ± SD. ^#^*p*<0.05, ^##^*p*<0.01 vs. sham group or normal group.

Serum, plasma, and urine samples collected from the MI mice were performed with metabolomics analysis to systematically excavate metabolites with significant changes at the early stage of myocardial ischemia. A definite separation among sham, model-2w, and model-3w groups was observed in the PCA and OPLS-DA patterns in serum and urine samples **(Supplementary Figure S5)**. A total of 52 differential metabolites (VIP > 1 and p-value < 0.05) were detected from the comparison of sham versus MI mice (model 2-3w) **(Table 1)**. There was a distinct change of metabolites in various body fluids of MI mice. The metabolic pathways related to myocardial ischemia were further revealed based on screening results of the above differential metabolites, respectively **(Figure 1A)**.

### Xanthurenic acid was identified as the metabolic marker of myocardial ischemia

In addition to a majority of biomarkers known to be closely associated with MI, such as urea, taurine, and methionine, some new biomarkers were also discovered and showed different changes in the early stage of MI **(Figure 1B)**. The metabolites xanthurenic acid, 2-arachidonoylglycerophoshphocholine, 7-methylguanine, and fructoselysine were subsequently performed for targeted analysis using a tandem LC-MS-based approach in MI mice. The levels of metabolites xanthurenic acid and 2-arachidonoylglycerophoshphocholine were significantly increased only 1 day after myocardial ischemia in mice **(Figure 1C)**. And then, the change of these two metabolites was further verified in clinical MI patients and normal healthy persons **(Figure 1D)**. Additionally, ROC analyses were used to assess the specificity and sensitivity of xanthurenic acid and 2-arachidonoylglycerophoshphocholine in the clinical diagnosis of MI, the results showed that xanthurenic acid had a higher AUC value and a better correlation with MI compared with 2-arachidonoylglycerophoshphocholine **(Figure 1E)**.

### Elevation of xanthurenic acid levels aggravated myocardial ischemia injury

**Figure 2:**
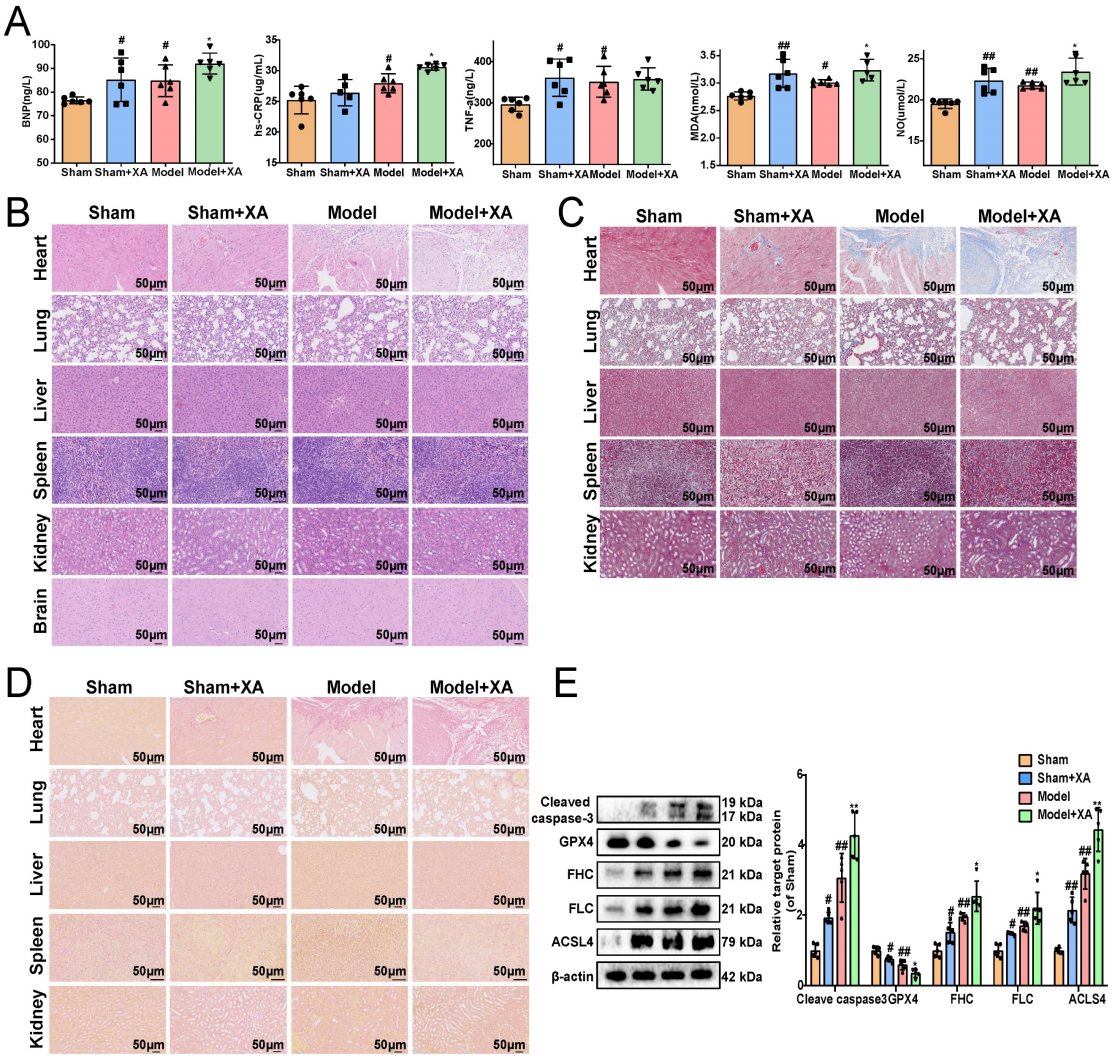
** Elevation of xanthurenic acid level aggravates myocardial ischemia injury. A.** Serum biochemical indicators contents include BNP, hs-CRP, TNF-α, MDA, and NO in sham and MI model groups mice administered xanthurenic acid (XA), and were detected by ELISA (*i.p.* 100 mg/kg) (n = 6). **B-D.** Histological analysis of heart, lung, liver, spleen, kidney, and brain slices by hematoxylin and eosin (H&E), masson's trichrome, and sirius red staining (n = 5) (All images were 200X magnification except for spleen was 400X magnification; the lines marked in all figures represent 50 µm). **E.** Western blots and quantitative results of cleaved caspase-3, GPX4, FHC, FLC, and ACSL4 in each group mice heart (n = 5). Data are presented as mean ± SD. ^#^*p* < 0.05, ^##^*p* < 0.01 vs. sham group, ^*^p < 0.05, ^**^p < 0.01 vs. model group.

To further revealed the association between the elevated levels of xanthurenic acid and MI, xanthurenic acid was injected intraperitoneally in mice. We found that the elevation of xanthurenic acid levels further increased the content of BNP, hs-CRP, MDA, TNF-α, and NO in the serum of MI mice **(Figure 2A)**. Histopathological examination results showed that treatment with xanthurenic acid for 8 days could lead to cardiomyocyte necrosis, myocardial fibrosis, and inflammatory cell infiltration in the heart tissue of mice with MI. We also observed that elevated levels of xanthurenic acid slightly increased the morphological damage and fibrosis of lung tissue in mice **(Figure 2B-D, Supplementary Figure S6A-C)**. However, treatment with xanthurenic acid has no significant impact on other major organs. Besides, the elevation of xanthurenic acid was found to enhance the expression of FHC, FLC, ACSL-4, and cleaved caspase-3, and reduce GPX4 expression, which demonstrated its effect closely associated with the activation of ferroptosis and apoptosis in MI mice **(Figure 2E)**.

### KMO overexpression is a key factor leading to the elevation of xanthurenic acid in myocardial ischemia

**Figure 3:**
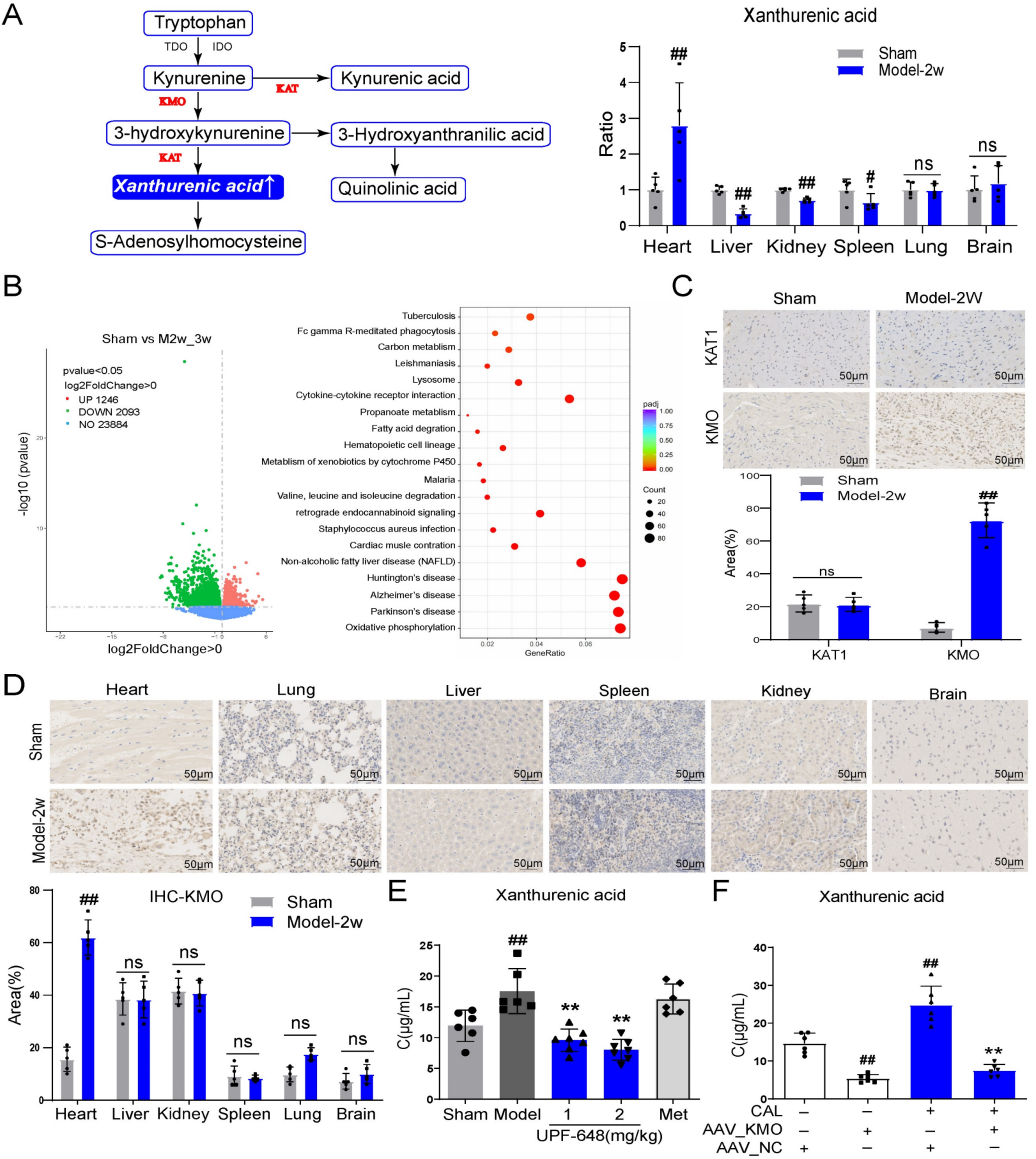
** KMO overexpression is a key factor leading to the elevation of xanthurenic acid in MI. A.** The level of xanthurenic acid in the different tissues of MI mice after 2 weeks' CAL (n = 5). Xanthurenic acid is one of the metabolites involved in the tryptophan metabolic pathway and the content of xanthurenic acid in the tissues were quantitatively analyzed by LC-MS. **B.** The heart tissues of sham and MI model groups mice were collected for transcriptome analysis (Sham: n = 3, Model 2-3w: n = 6). Volcano plot and KEGG pathway enrichment analysis of differential genes between sham, model 2-3w. Red dots in volcanoes represent genes whose expression is up-regulated, and green dots in volcanoes represent genes whose expression is down-regulated. **C.** The expressions of KAT1 and KMO in heart tissues of sham and MI model groups mice, and were determined by the immunohistochemical staining (n = 5) (400X magnification, the lines marked in all figures represent 50 µm). **D.** The expression level of KMO in the different tissues of sham and MI model groups mice after 2 weeks' CAL, and were measured by immunohistochemical staining (n = 5) (400X magnification, the lines marked in all figures represent 50 µm). Image J software was used for area statistics. **E.** Effects of KMO inhibitor UPF-648 on the contents of xanthurenic acid in urine samples of MI model groups mice after 2 weeks' CAL (n = 6-7) (1 mg/kg/day and 2 mg/kg/day). **F.** Effects of cardiac-specific knockdown of KMO expression through infection of recombinant AAV-KMO on the contents of xanthurenic acid in urine samples of MI model groups mice after 2 weeks' CAL (n = 6). Data are presented as mean ± SD. ^#^*p* < 0.05, ^##^*p* < 0.01 vs. sham or sham + AAV-NC group, ^*^p < 0.05, ^**^p < 0.01 vs. model or model + AAV-NC group; ns means no significance.

Xanthurenic acid is mainly involved in the metabolism of tryptophan. To explore the tissue specificity of xanthurenic acid, the level of xanthurenic acid was measured in six kinds of organs. Interestingly, the results indicated that xanthurenic acid increased most significantly in heart tissue** (Figure 3A)**. Based on the above results, we further screened specific genes of MI mice in the heart tissues by transcriptomics. A total of 1249 differential genes were screened between MI mice and the sham operation group, as shown in **Figure 3B**. The top 50 differential genes obtained from the comparison of MI (CAL 2-3w) and sham mice were shown in **Table 2**. The analysis of KEGG enrichment of different genes revealed that abnormal energy metabolism and oxidative stress mainly occurred in MI mice **(Figure 3B)**.

Based on the above transcriptome results, the related regulatory enzymes of xanthurenic acid were further investigated. Transcriptional data from cardiac tissue revealed that KMO and kynurenine aminotransferase 1 (KAT1) changed prominently in MI mice (CAL 2-3w). It's worth noting that, KMO appeared in the top 10 differential genes from the comparison of sham versus MI mice **(Table 2)**. Meanwhile, immunohistochemical results of heart tissues showed that the expression of KMO dramatically increased in MI mice, while KAT1 expression did not change **(Figure 3C)**. Combined with the results of gene and protein expression, KMO might be the key regulatory metabolic enzyme leading to the abnormal elevation of xanthurenic acid in MI. Moreover, we further found that the expression of KMO only profoundly elevated in the heart tissues of MI mice, while no obvious impacts were observed in other major organs **(Figure 3D)**. Additionally, the inhibitor of KMO (UPF-648) and recombinant AAV-KMO were further adopted and the results showed that KMO inhibition could effectively prevent the abnormal increase of xanthurenic acid **(Figure 3E-F)**. All these data suggested that the primary cause of increased metabolite xanthurenic acid in MI might be the overexpression of KMO.

### Inhibition of KMO effectively alleviated myocardial ischemia injury

Firstly, the inhibitor of KMO (UPF-648) was used to explore the role of KMO *in vitro* and *in vivo*. We found that UPF-648 could successfully inhibit the expression of KMO **(Supplementary Figure S7A-B)**, and it could effectively increase the cell viability as well as decrease the LDH release in OGD-induced cardiomyocytes injury **(Supplementary Figure S8A-B)**. Meanwhile, UPF-648 could ameliorate the histological features and reduce the serum level of BNP, hs-CRP, MDA, nitric oxide (NO), and TNF-α in MI mice **(Supplementary Figure S9A-B)**. Also, the transmission electron microscopy results showed that UPF-648 could significantly alleviate myocardial ultrastructure damage, reduce cellular edema and promote the maintenance of the mitochondrial matrices **(Supplementary Figure S9C).**

**Figure 4:**
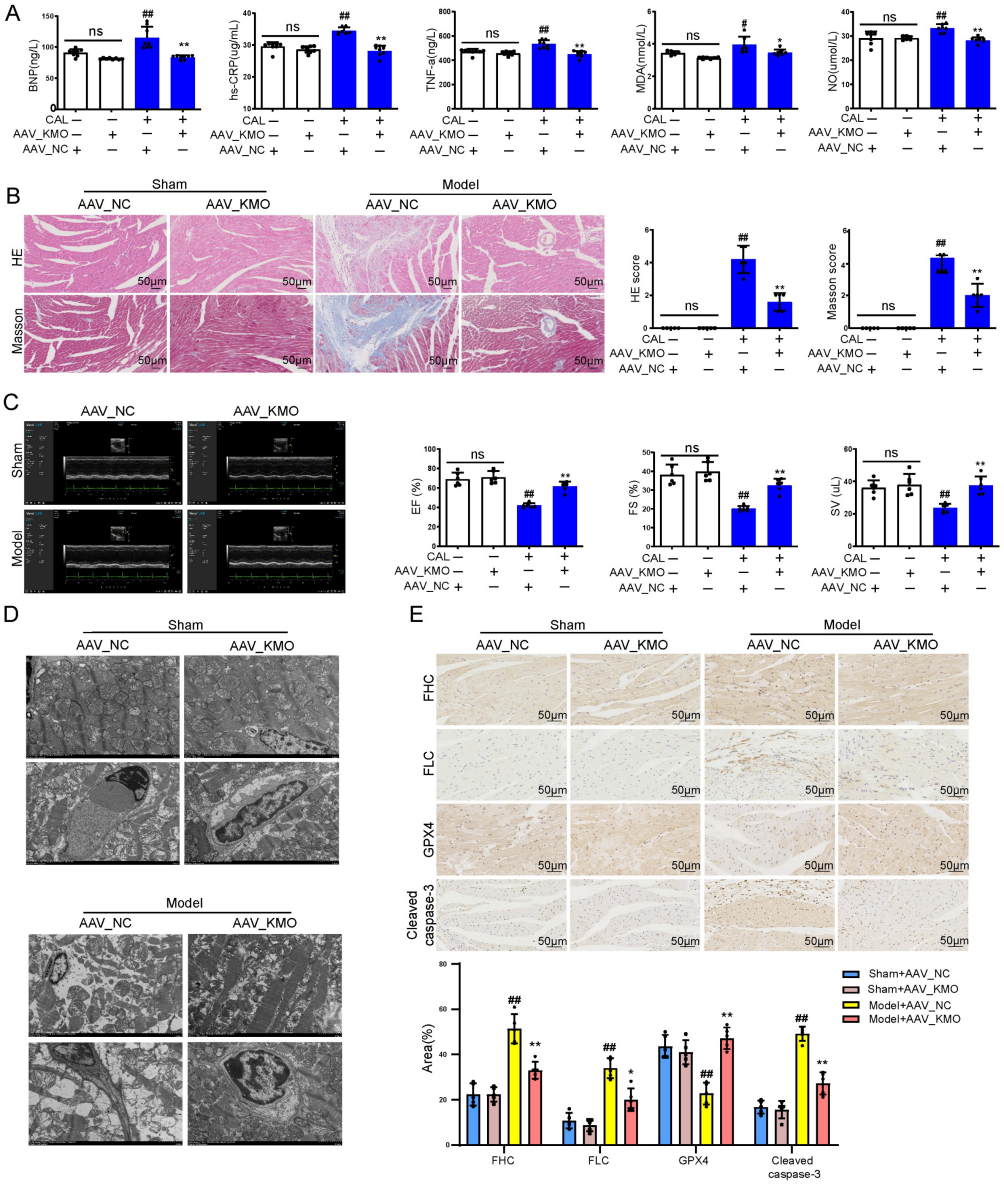
** Cardiac-specific knockdown of KMO effectively alleviates myocardial injury in MI. A.** Serum biochemical indicators contents include BNP, hs-CRP, TNF-α, MDA, and NO in sham and MI model groups mice with cardiac-specific knockdown of KMO expression and were detected by ELISA (n = 7-8). **B.** Histological analysis of heart slices was shown by H&E and Masson's trichrome staining in sham and the MI model groups mice with cardiac-specific knockdown of KMO expression (n = 5) (200X magnification, scale bar, 50 µm). **C.** Representative echocardiographic graphs are shown. The echocardiographic parameters were measured, including ejection fraction (EF), fractional shortening (FS), and stroke volume (SV) in sham and the MI model groups mice with cardiac-specific knockdown of KMO expression (n = 6). **D.** Representative transmission electron microscope images of heart tissues with cardiac-specific knockdown of KMO expression (n = 3). **E.** Representative immunohistochemical staining of FHC, FLC, GPX4, and cleaved caspase-3 in sham and MI model groups mice heart with cardiac-specific knockdown of KMO expression (n = 5) (400X magnification, scale bar, 50 µm). Data are presented as mean ± SD. ^#^*p* < 0.05, ^##^*p* < 0.01 vs. sham + AAV-NC group, ^*^p < 0.05, ^**^p < 0.01 vs. model + AAV-NC group; ns means no significance.

Then cardiac-specific KMO knockdown mice were constructed to further explore the function of KMO in MI injury. Inhibition of cardiac expression of KMO was achieved by intramyocardial injection of AAV9 loading the specific sequence targeting KMO (AAV-KMO) or an AAV-negative control (AAV-NC) in mice. Immunohistochemical analysis confirmed that the knockdown rate of KMO in heart tissue was over 50% **(Supplementary Figure S7C)**. Based on the above results, KMO inhibition profoundly ameliorated the biochemical indexes (BNP, hs-CRP, MDA, NO, and TNF-α) associated with myocardial damage in serum (**Figure 4A**), as well as improved the pathological damage of myocardial tissue** (Figure 4B)**. What's more, it showed an evident effect on improvement in cardiac function, such as LV ejection fraction, LV fractional shortening, and stroke volume** (Figure 4C, Supplementary Table S6)**. We also found that inhibition of KMO in MI mice significantly ameliorated myocardial ultrastructure damage and mitochondrial morphology** (Figure 4D)**. Moreover, we found that KMO inhibition could significantly inhibit the activation of ferroptosis and apoptosis in MI injury **(Figure 4E)**. All these findings suggested that KMO inhibition could observably improve cardiac performance in MI injury.

### Inhibition of KMO prevents myocardial damage by maintaining mitochondrial fusion and fission balance

**Figure 5:**
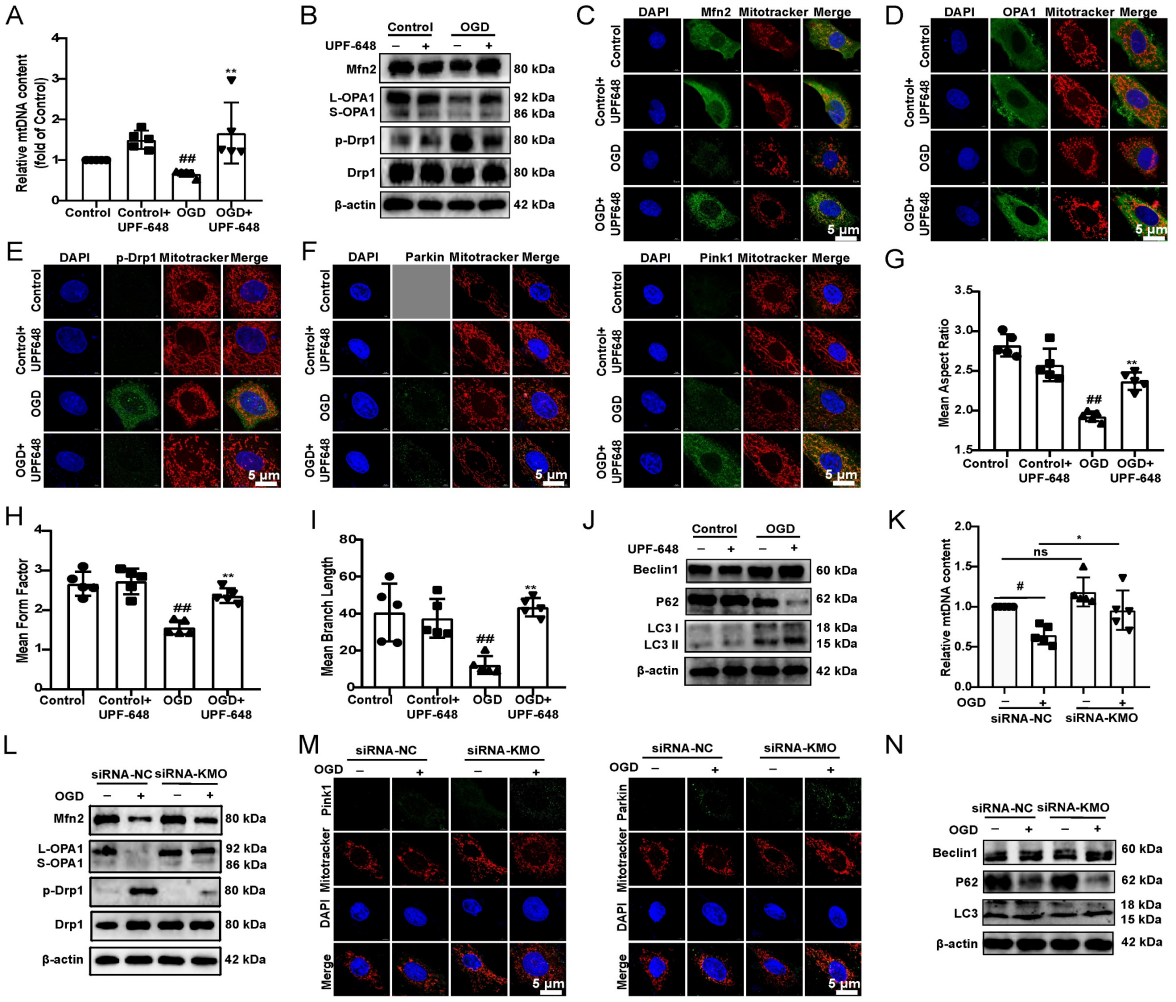
**Inhibition of KMO reduces OGD-induced cardiomyocyte injury by regulating mitochondrial fission/fusion. A.** RT-PCR analysis of mtDNA content in the OGD-induced H9c2 cells injury model treated with KMO inhibitor UPF648 (2 µmol/L) (n = 5).** B**. Western blotting analysis of Mfn2, OPA1, p-Drp1, and Drp1 in the OGD-induced H9c2 cells injury model treated with KMO inhibitor UPF648 (2 µmol/L) (n = 5). **C - F**. Representative immunofluorescence images of Mfn2, OPA1, p-Drp1, Parkin, and Pink1 in the OGD-induced H9c2 cells injury model treated with KMO inhibitor UPF648 (2 µmol/L) (n = 5). **G-I.** The average aspect ratio and form factor of the mitochondria labeled with MitoTracker® Deep Red were measured. Smaller mitochondrial length, aspect ratio, and form factor values represent increased mitochondrial fragmentation (n = 5). **J.** Western blotting analysis of Beclin1, P62, and LC3I/II in the OGD-induced H9c2 cells injury model treated with KMO inhibitor UPF648 (2 µmol/L) (n = 5). **K.** RT-PCR analysis of mtDNA content in the OGD-induced H9c2 cells injury model treated with siRNA-KMO or siRNA-NC (n = 5). **L**. Western blotting analysis of Mfn2, OPA1, p-Drp1, and Drp1 in the OGD-induced H9c2 cells injury model treated with siRNA-KMO or siRNA-NC (n = 5). **M**. Representative immunofluorescence images of Parkin, and Pink1 in the OGD-induced H9c2 cells injury model treated with siRNA-KMO or siRNA-NC (n = 5). **N**. Western blotting analysis of Beclin1, P62, and LC3I/II in the OGD-induced H9c2 cells injury model treated with siRNA-KMO or siRNA-NC (n = 5). Data are presented as mean ± SD. ^#^*p* < 0.05, ^##^*p* < 0.01 vs. control group or control + siRNA-NC group, ^*^p < 0.05, ^**^p < 0.01 vs. OGD group or OGD + siRNA-NC group; ns means no significance.

Next, we investigated the specific mechanisms of KMO inhibition in improving MI injury *in vitro* and *in vivo*. Previous researches reported that KMO is a protein localized to the outer mitochondrial membrane 34. Thus, this study first investigated the effect of inhibition of KMO on mitochondrial function. The detection of mitochondrial copy number found that KMO inhibitors significantly reversed the trend of OGD-induced reduction in mtDNA number **(Figure 5A)**. Meanwhile, the KMO inhibitor markedly up-regulated the expression of OPA1, Mfn2 and inhibited phosphorylation of Drp1 (Ser616) in OGD-induced cardiomyocytes injury and MI injury **(Figure 5B-E, Supplementary Figure S10 and Figure S11A-B)**. And the inhibition of KMO further activated the expression of mitochondrial autophagy Pink1 and Parkin in OGD-induced cardiomyocytes injury** (Figure 5F, Supplementary Figure S11C).**

**Figure 6:**
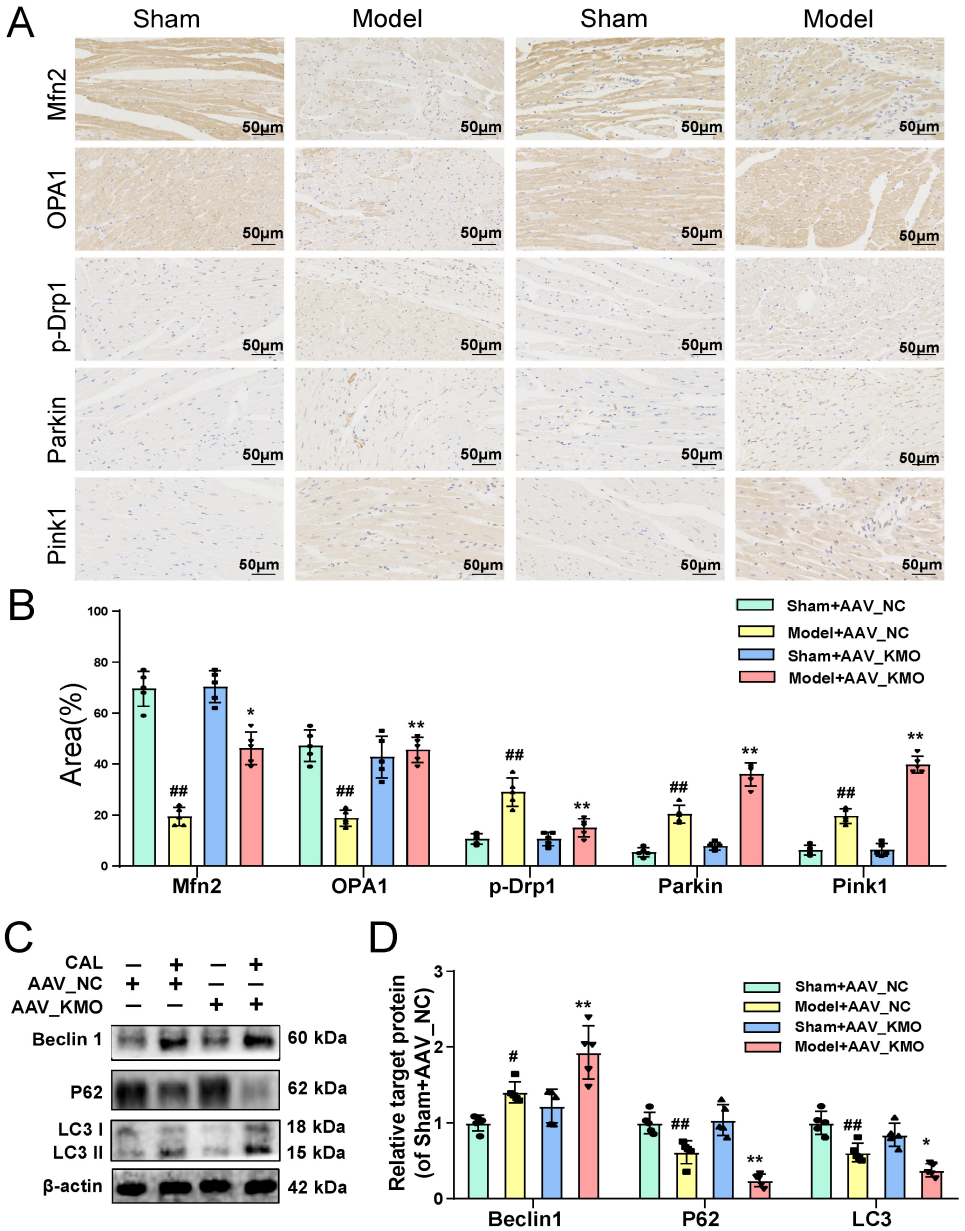
** Cardiac-specific knockdown of KMO prevents myocardial injury via maintaining mitochondrial fusion/fission balance. A-B.** The expressions of Mfn2, OPA1, p-Drp1, Parkin, and Pink1 were detected by immunohistochemistry in heart tissue of sham and MI model groups mice with cardiac-specific knockdown of KMO expression (n = 5) (400X magnification, scale bar, 50 µm). **C-D.** Western blots and quantitative results of Beclin1, P62, and LC3I/II in heart tissue of sham and MI model groups mice with cardiac-specific knockdown of KMO expression (n = 5). Data are presented as mean ± SD.^ #^*p* < 0.05, ^##^*p* < 0.01 vs. sham + AAV-NC, ^*^p < 0.05, ^**^p < 0.01 vs. model + AAV-NC; ns means no significance.

Besides, the results of immunofluorescence further indicated that the inhibitor of KMO (UPF-648) evidently ameliorated the fragmentation of mitochondria in the OGD injury model **(Figure 5G-I)**. Interestingly, the KMO inhibition also caused an increase in its downstream autophagy proteins LC3II and Beclin1 and decreased expression of P62 **(Figure 5J, Supplementary Figure S11D)**. Moreover, KMO-siRNA not only rescued the reduction in mtDNA content caused by OGD, also prevented OGD-induced cardiomyocytes injury by maintaining mitochondrial fusion/fission balance **(Figure 5K-N, Supplementary Figure S11E-G)**. Meanwhile, we further validated these findings in CAL-induced MI injury using cardiac-specific knockdown of KMO. The result indicated that cardiac-specific knockdown of KMO promoted the fusion/fission balance of mitochondria and further accelerated the progress of autophagy to reduce damaged mitochondria** (Figure 6)**. All the above data suggested that maintaining mitochondrial fusion and fission balance is indeed an important way for KMO inhibition in preventing myocardial ischemia injury.

### Ginsenoside Rb3 was screened as a novel KMO inhibitor and ameliorated myocardial ischemia injury

**Figure 7:**
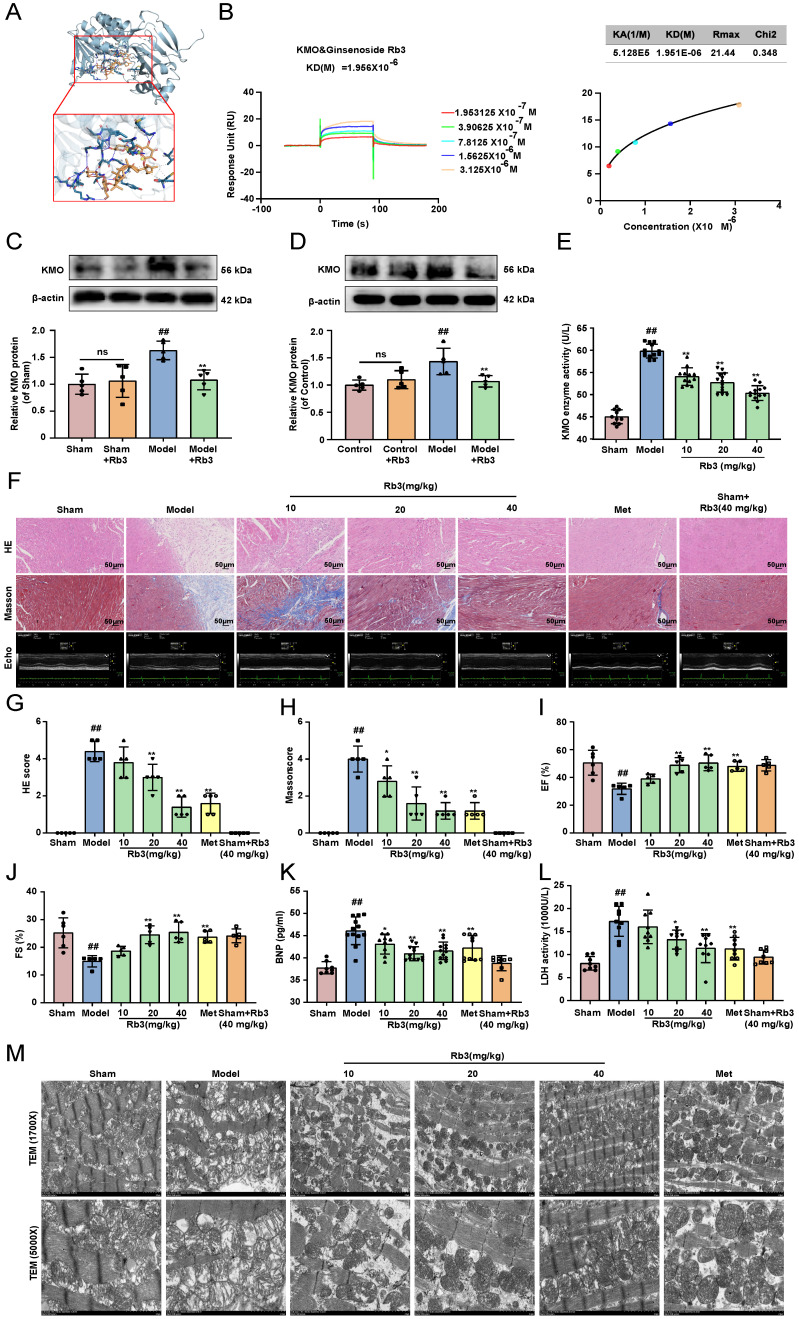
** Ginsenoside Rb3 is screened as a novel KMO inhibitor and ameliorated cardiac injury in MI. A.** A molecular docking model carried out by Autodock vina revealed that ginsenoside Rb3 bonded to the KMO. **B.** SPR analysis of the binding affinity of ginsenoside Rb3 and KMO protein. Apparent equilibrium dissociation constants (Kd) were calculated as the ratio of Kd/Ka. The Kd (mol/L) value between Ginsenoside Rb3 and KMO was 1.951x10^-6^ mol/L. **C-D.** Representative western blot results in the analysis of KMO in heart tissues of MI model group mice and H9c2 cells (n = 5). **E.** The enzyme activity of KMO in sham and MI model group mice treated with ginsenoside Rb3 (n = 10-13, one-way ANOVA followed by Bonferroni post hoc test). **F-J.** HE staining and Masson staining cross-sections of heart tissues (n = 5) (200X magnification, scale bar, 50 µm) and representative echocardiographic graphs were shown (n =5-6), respectively. **K-L.** Serum biochemical indicators contents include BNP and LDH in sham and MI model groups mice treated with ginsenoside Rb3, and were detected by ELISA (n = 8-12). **M.** Representative transmission electron microscope images of heart tissues in sham and MI model groups mice treated with ginsenoside Rb3 (n = 3). Data are presented as mean ± SD. ^#^*p* < 0.05, ^##^*p* < 0.01 vs. sham or control group, ^*^p < 0.05, ^**^p < 0.01 vs. model or OGD group; ns means no significance.

The above results suggested that KMO might serve as a potential target for the treatment of MI injury, thus, molecular docking technology was further conducted to screen novel KMO inhibitors based on the Traditional Chinese Medicine Systems Pharmacology Database and Analysis Platform. The virtual screening results were sorted according to the predicted binding free energy, which ranged from -5.8 to -13.0 kcal/mol. Protein-ligand interaction analysis showed that hydrogen bonds and hydrophobic interaction were the main modes of interaction between these compounds and KMO. In these compounds, Ginsenoside Rb3 showed the strongest binding affinity with human KMO, suggesting that ginsenoside Rb3 might be a new potential KMO inhibitor** (Table 3)**. Meanwhile, the molecular docking results revealed that ginsenoside Rb3 bound to the binding pocket, which might form hydrophobic interactions with Arg40, Ser53, and Gln283 of KMO (the docking pose was shown in **Figure 7A**). To further confirm whether ginsenoside Rb3 directly targeted KMO, the surface plasmon resonance (SPR) technology was performed. Ginsenoside Rb3 exhibited a strong binding affinity for KMO with an estimated equilibrium dissociation constant of 1.956 μM **(Figure 7B)**. Besides, the interaction pattern between compounds with the top 5 affinities of KMO except for ginsenoside Rb3 was shown in **Supplementary Figure S13**. Subsequently, the inhibitory effect of ginsenoside Rb3 on KMO was verified *in vivo* and *in vitro.* The results suggested that the expression and enzymatic activity of KMO were significantly inhibited by ginsenoside Rb3 in both myocardial tissues and cardiomyocytes **(Figure 7C-E)**.

We next investigated the effect of ginsenoside Rb3 on the myocardial ischemia-induced cardiac injury. Ginsenoside Rb3 was orally administrated at doses of 10, 20, and 40 mg/kg/day for 8 days after CAL for one week. As illustrated in **Figure 7F-J**, ginsenoside Rb3 could effectively improve cardiac pathological injury and functions (LV ejection fraction and LV fractional shortening), as well as significantly ameliorate the biochemical indexes (BNP and LDH) related to myocardial injury in serum **(Figure 7K-L)**. Simultaneously, it also exhibited a prominent effect on ameliorating myocardial ultrastructure damage and mitochondrial morphology **(Figure 7M)**. In addition, ginsenoside Rb3 could also significantly increase cell viability in the OGD-induced cardiomyocytes injury **(Supplementary Figure S8C)**. All the above results indicated that ginsenoside Rb3 may be a novel KMO inhibitor and is effective in improving the cardiac damage of MI.

### Ginsenoside Rb3 attenuated myocardial ischemia injury through regulating KMO mediated mitochondrial fusion and fission

**Figure 8:**
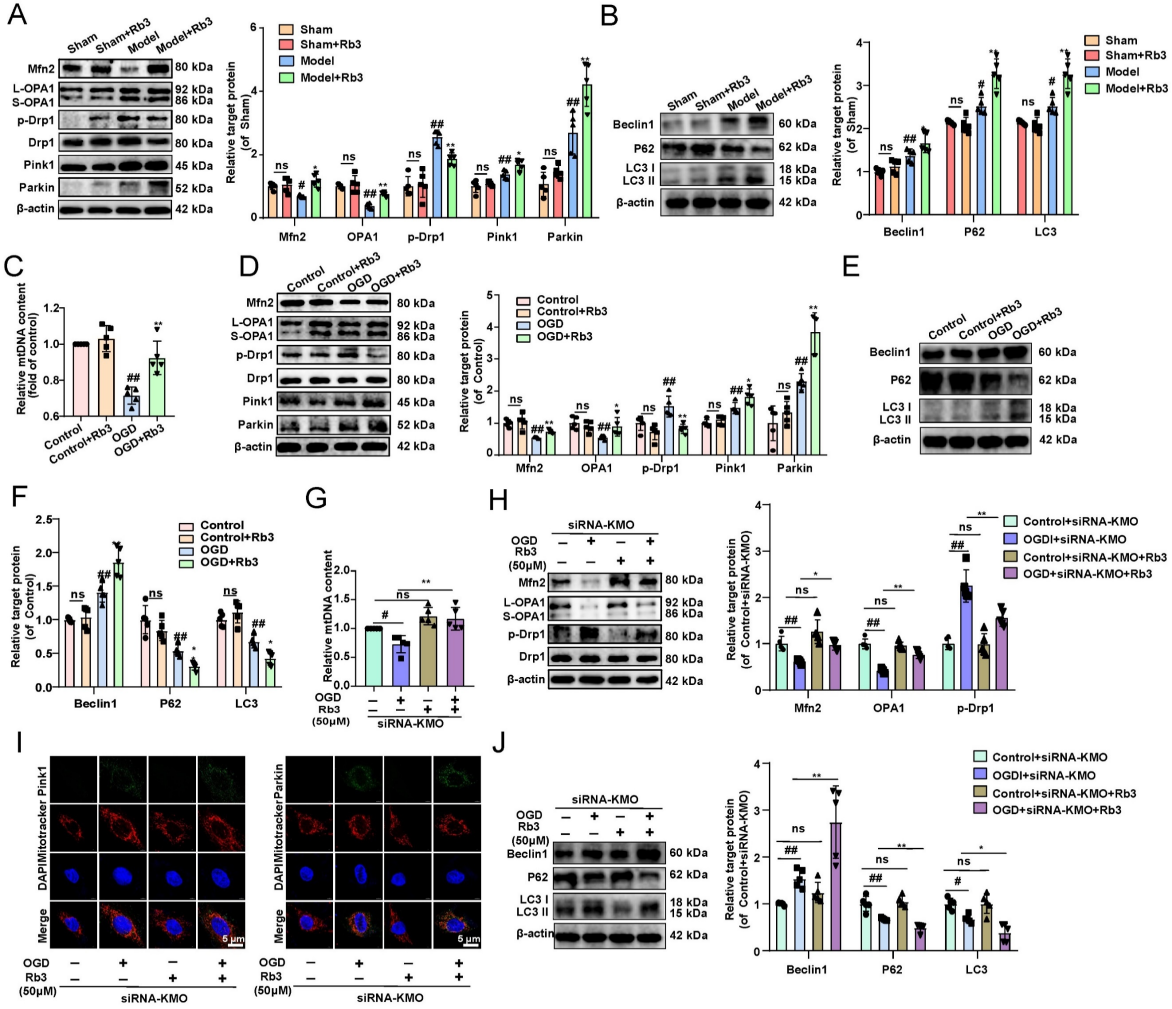
** Ginsenoside Rb3 attenuates MI through regulating KMO mediated mitochondrial fusion/fission. A.** Western blots and quantitative results of Mfn2, OPA1, p-Drp1, Drp1, Parkin, and Pink1 in sham and MI model groups mice treated with ginsenoside Rb3 (n = 5). **B.** Western blots and quantitative results of Beclin1, P62, and LC3I/II in sham and MI model groups mice treated with ginsenoside Rb3 (n = 5). **C.** RT-PCR analysis of mtDNA content in the OGD-induced H9c2 cells injury model treated with ginsenoside Rb3 (n = 5). **D.** Western blots and quantitative results of Mfn2, OPA1, p-Drp1, Drp1, Parkin, and Pink1 in the OGD-induced H9c2 cells injury model treated with ginsenoside Rb3 (n = 5). **E-F.** Western blots and quantitative results of Beclin1, P62, and LC3I/II in the OGD-induced H9c2 cells injury model treated with ginsenoside Rb3 (n = 5). **G.** RT-PCR analysis of mtDNA content in the OGD-induced H9c2 cells injury model treated with siRNA-KMO or ginsenoside Rb3 (n = 5). **H.** Western blots and quantitative results of Mfn2, OPA1, p-Drp1, and Drp1 in the OGD-induced H9c2 cells injury model treated with siRNA-KMO or ginsenoside Rb3 (n = 5). **I.** Representative immunofluorescence images of Pink1 and Parkin in the OGD-induced H9c2 cells injury model treated with siRNA-KMO or ginsenoside Rb3 (n = 5). **J.** Western blots and quantitative results of Beclin1, P62, and LC3I/II in the OGD-induced H9c2 cells injury model treated with siRNA-KMO or ginsenoside Rb3 (n = 5). Data are presented as mean ± SD. ^#^*p* < 0.05,^ ##^*p* < 0.01 vs. sham or control or control + siRNA-KMO group, ^*^p < 0.05, ^**^p < 0.01 vs. model or OGD or OGD + siRNA-KMO group; ns means no significance.

What's more, the effect of ginsenoside Rb3 on mitochondrial fusion and fission was investigated *in vitro* and *in vivo*. And we found that ginsenoside Rb3 could significantly reversed the trend of OGD-induced reduction in mtDNA number, as well as increase the expression of Mfn2, and OPA1 and decrease the phosphorylation of Drp1 in OGD-induced cardiomyocytes injury and MI injury. In addition, mitochondrial autophagy proteins of Pink1 and Parkin as well as the downstream proteins Beclin1, P62, and LC3II were further altered by ginsenoside Rb3 administration** (Figure 8A-F, Supplementary Figure S14)**. These results consisted of the effect of the inhibitor of KMO and cardiac-specific KMO knockdown in MI mice. Besides, KMO-siRNA evidently enhanced the regulation of ginsenoside Rb3 on mtDNA number, mitochondrial fusion/fission and further promoted mitochondrial autophagy (**Figure 8G-J**). Taken together, all results indicated that ginsenoside Rb3 could attenuate MI injury through maintaining mitochondrial fusion and fission balance and KMO might exert a critical role in its effect.

## Discussion

In our present study, high throughput metabolomics analysis showed that xanthinic acid may be a new metabolic marker of myocardial ischemia. Furthermore, the combined metabolic and transcriptional data demonstrated that KMO is a key enzyme regulating the abnormal elevation of xanthurenic acid in MI. Pharmacological or heart-specific inhibition of KMO significantly attenuated OGD-induced cardiomyocytes injury, as well as ligation induced myocardial ischemic injury.

Early identification and intervention of MI could delay the progression into myocardial infarction, heart failure, and some other cardiovascular diseases. It is of great significance to find relevant biomarkers for clinical diagnosis and treatment. Evaluation of myocardial pathological injury and cardiac function at different ischemic time points showed that significant myocardial injury occurred around 2-3 weeks after ischemia. And our study further revealed systemic metabolic alterations and constructed a metabolic network map of MI through untargeted metabolomics. Significant changes were found in urea, taurine, hemolysase, niacin, xanthurenic acid, 2-arachidonylglycerophospcholine, fructose-lysine, and 7-methylguanine in MI mice. Among them, urea 35, 36, LysoPE 37, 38, niacin 39, and taurine 40 have been clinically reported to be highly associated with myocardial infarction and other cardiovascular diseases. And it also included many metabolites whose specific functions in MI are still unclear, such as xanthurenic acid, 2-arachidonylglycerophospcholine, 7-methylguanine, fructoysine, etc. Additionally, targeted metabolomics was conducted to identify potential metabolic markers in MI mice and patients. And we found that both xanthurenic acid and 2-arachidonylphosphocholine significantly elevated at the early stage after MI injury. This is basically consistent with the target verification results of MI clinical specimens. Then, the sensitivity and specificity of these two potential metabolic markers were evaluated, and xanthurenic acid showed great potential as a diagnostic marker for MI.

Xanthurenic acid is mainly involved in the tryptophan/cyuridine metabolic pathway associated with neuropsychiatric and immune diseases. In recent years, aberrant activation of the Trp/Kynurenine pathway has been found to be highly associated with inflammation and cardiovascular diseases 41, 42. Of note, 3-hydroxykynurenine/xanthurenic acid was associated with increased mortality in patients with heart failure 43. However, it is unclear how xanthurenic acid participates in the pathologic processes of MI. To investigate the function of xanthurenic acid *in vivo*, its effects on six major organs were observed. And we found that elevation of xanthurenic acid significantly induced myocardial injury and fibrosis in sham mice but had no obvious effect on other organs. To further explore how xanthurenic acid promoted myocardial injury, we investigated its effects on two important cardiomyocyte death pathways, including apoptosis and ferroptosis 44. And the results suggested that high xanthurenic acid levels could trigger myocardial injury and aggravate the pathology of myocardial infarction, which might be related to the activation of cardiomyocyte apoptosis and ferroptosis.

Based on the above results, transcriptomic analysis was further carried out to screen out the specific gene clusters of MI and to further explore the key regulatory enzymes of xanthurenic acid in heart tissue. Enrichment analysis showed that MI-specific genes were mainly involved in oxidative phosphorylation, tricarboxylic acid cycle (TCA), electron transport chain, and nucleoside triphosphate metabolism. Oxidative phosphorylation occurs mainly in the inner mitochondrial membrane of eukaryotic cells or the cytoplasm of prokaryotic cells during biochemical processes. It is the energy released by substances in the body through the oxidation process of the respiratory chain and supplies ADP and inorganic phosphate to synthesize ATP 45, which is consistent with TCA 46, and both play a key role in the body's energy metabolism. And our results indicated that abnormal energy metabolism mainly occurred in myocardial ischemia. Combined with previous transcriptome data and the expression results in different tissues of MI mice, we found that KMO expression significantly increased only in heart tissues. Meanwhile, further application of KMO inhibitors and AAV technology demonstrated that KMO inhibition significantly reduced the increase of xanthurenic acid *in vivo*. These results suggested that the significant increase in KMO expression might be a key factor in the abnormal increase of xanthurenic acid content in MI.

Previous studies have shown that KMO is a potential therapeutic target for several neurological diseases, such as Alzheimer's disease (AD) and Huntington's disease (HD) 47, 48. But the relationship between KMO and MI injury is unclear. This study firstly demonstrated that KMO inhibition effectively ameliorated myocardial ischemia injury. Interestingly, heart-specific inhibition of KMO significantly prevented myocardial ferroptosis and apoptosis. These results further confirmed that KMO might serve as a new therapeutic target for MI injury. KMO is a protein specifically localized on the outer mitochondrial membrane (OMM) 49. Over-activation of KMO has been found to induce ROS production and mitochondrial dysfunction in human primary neurons 50. Notably, mitochondrial dysfunction is a key pathological mechanism in cardiovascular diseases, especially in the development of MI injury. The majority (about 95%) of ATP consumed by the heart comes from oxidative metabolism in mitochondria, which accounts for about 1/3 of the volume of adult cardiomyocytes 51. Meanwhile, a previous study has found that KMO was involved in the regulation of mitochondrial dynamics and mitochondrial autophagy in Drosophila melanogaster under physiological conditions 34. Therefore, we investigated the effect of KMO inhibition on mitochondrial fission and fusion. And we found that the expression of Drp1 (pSer616) decreased, and the expression of Mfn2 and L-OPA1/S-OPA1 markedly increased after KMO inhibition, which suggested that KMO inhibition prevented mitochondrial excessive fission and promoted mitochondrial fusion. Modest mitochondrial fission inhibition and accelerated mitochondrial fusion have been shown to protect the heart from ischemic damage 52.

What's more, the imbalance of mitochondrial fusion and fission leading to mitochondrial fragmentation is the premise of mitochondrial autophagy 53, 54. In mammals, Pink1/Parkin pathway is a classical way to regulate mitochondrial autophagy 55. The activation of the Pink1/Parkin pathway could promote downstream autophagosomes to bind to damaged mitochondria, thereby clearing these mitochondria 56, 57. Subsequently, inhibition KMO has been demonstrated to significantly activate the Pink1/Parkin pathway of mitophagy, as well as accelerate the occurrence of its downstream autophagy. Above results indicated that KMO inhibition was essential in the protection of myocardial ischemia injury by maintaining mitochondrial fusion and fission balance.

Given the critical role of KMO in myocardial ischemia injury, potential KMO inhibitors were further screened by molecular docking and SPR techniques. And we found that ginsenoside Rb3 had a certain binding affinity with KMO, and its inhibitory effect on KMO was verified. Ginsenoside Rb3, the main active component in ginseng, has been widely used to treat cardiovascular diseases 58, 59. Recent studies have reported that ginsenoside Rb3 can regulate mitochondrial function and exert anti-apoptotic and antioxidant effects 13, 59. Regarding the role of KMO in MI, whether ginsenoside Rb3 could exert cardioprotective effect via KMO remained to be further elucidated. And we observed that ginsenoside Rb3 significantly inhibited mitochondrial fission and accelerated mitochondrial fusion and autophagy, whereas, KMO inhibition could significantly enhance the effect of ginsenoside Rb3. These findings might indicate that KMO is essential or even a key functional protein for the cardioprotection of ginsenoside Rb3.

However, there are still some limitations in our current study. Only one metabolomics technology cannot cover all endogenous metabolites in organisms, and LC-MS alone may lose some key information. Therefore, it is necessary to further combine NMR, GC-MS, and other technologies to explain the metabolic characteristics of MI from a wider range of metabolic pathways. Additionally, many specific metabolites were selected, but only four were targeted for identification. Although key regulatory enzymes regulating the significant increase of XA were found, we only used inhibitors of KMO and AAV-shRNA to initially explore its heart-related function, and further cardiac conditional knockout mice are needed to confirm.

## Conclusion

**Figure 9:**
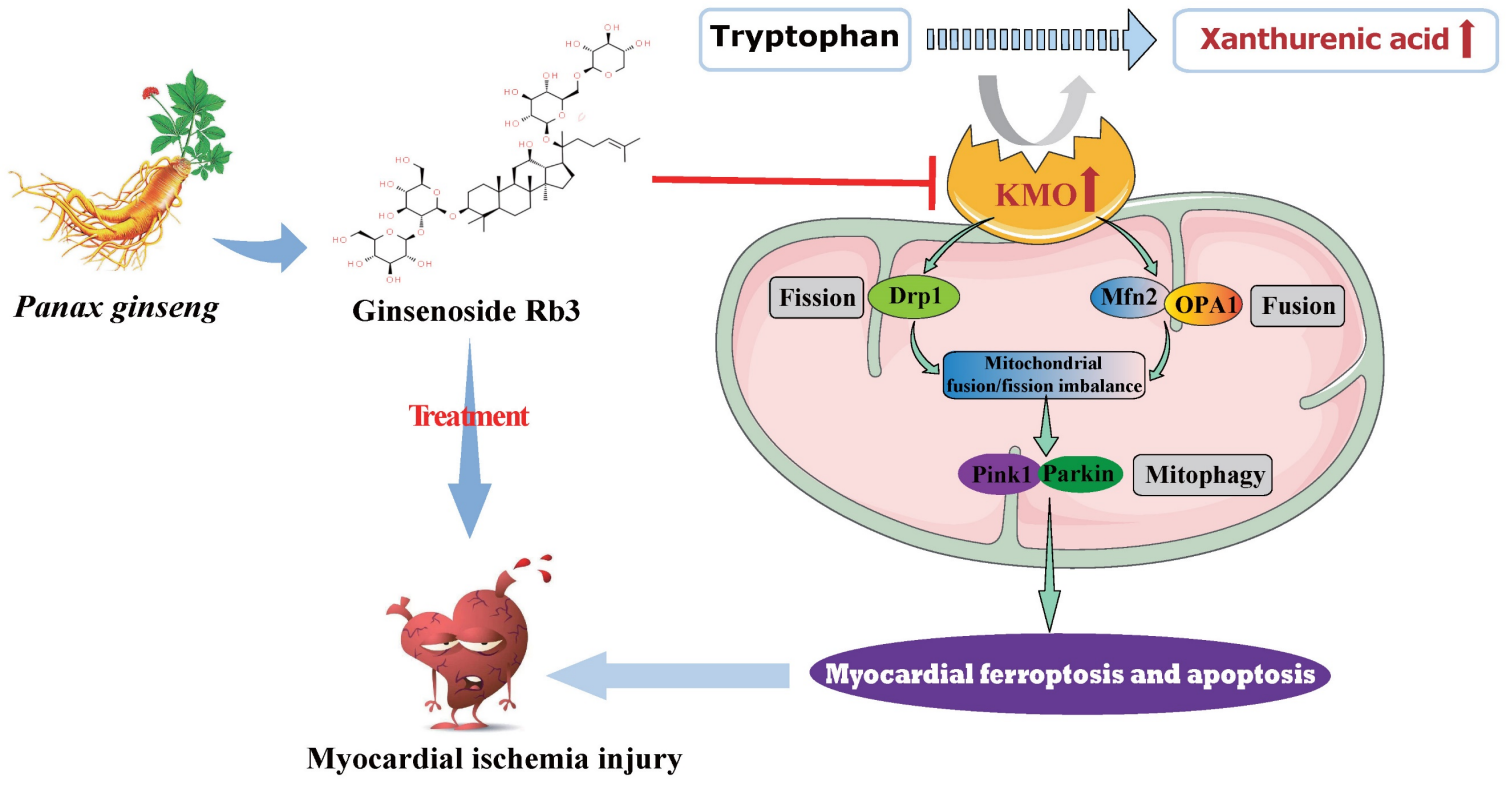
Schematic illustration of inhibition of KMO alleviated myocardial ischemic injury through regulating mitochondrial fission and fusion, and ginsenoside Rb3 was screened as a novel inhibitor of KMO.

In summary, metabolomics was adopted in this study to reveal metabolite signatures and the potential mechanisms closely related to MI injury, and four potential metabolic markers were verified at animal and clinical levels. Notably, we firstly identified the key role of xanthurenic acid in MI. What's more, we interestingly found that KMO overexpression was the key factor leading to the elevation of xanthurenic acid, and inhibition of KMO could effectively alleviate myocardial injury by regulating mitochondrial fission/fusion and appropriately activating the occurrence of autophagy. These observations will not only provide a novel metabolic marker for MI injury but also indicate that targeting KMO may represent an unrecognized therapeutic intervention for MI and other related cardiovascular diseases in the future. Meanwhile, ginsenoside Rb3 was screened as a novel inhibitor of KMO, and it was also found to have an excellent therapeutic effect on MI injury through regulating KMO mediated mitochondrial fusion and fission, which showed the great clinical potential to be seen as a candidate for the treatment of clinical MI **(Figure 9)**.

## Supplementary Material

Supplementary figures and tables.

## Figures and Tables

**Table 1:** Statistical analysis of 52 differential metabolites from the comparison of Sham versus MI mice

Trend	Sample	m/z	Rt(min)	VIP	p-value	FC	Identification
2-3W↑	Urine	162.1120	14.33	1.38	1.27E-12	2.92	L-isoleucyl-L-proline
		149.1066	4.46	1.37	2.70E-12	5.40	L-Isoleucine
		283.1393	15.72	1.36	2.74E-12	3.49	Prolylhydroxyproline
		283.1610	14.46	1.36	4.25E-10	2.58	L-Valine
		344.2272	2.58	1.35	2.69E-09	4.73	**Xanthurenic acid**
		136.0477	14.74	1.33	2.61E-07	7.37	Creatine
		213.1226	13.24	1.32	8.87E-07	14.18	L-prolyl-L-proline
		439.1370	10.34	1.30	1.65E-05	2.89	5-Methylcytidine
		371.2266	2.94	1.26	4.32E-06	5.82	3-Hydroxykynurenine
		555.2141	9.58	1.21	8.18E-04	2.66	7-Methylguanine
		148.0036	14.17	1.13	3.19E-04	7.65	Taurine
		259.1028	11.86	1.09	4.35E-04	3.43	Uridine
		332.1543	16.10	1.33	3.92E-05	1.91	N-Acetylglutamine
		200.1281	2.10	1.20	1.41E-03	1.66	5-Acetamidovalerate
		268.1538	1.99	1.19	2.14E-03	1.76	4-O-Methylmelleolide
	Serum	808.5825	24.83	5.52	2.67E-02	26.98	PC(16:0/22:5(7Z,10Z,13Z,16Z,19Z))
		522.3566	16.15	3.78	2.06E-02	1.64	2-Oleoylglycerophosphocholine
		786.6025	29.29	3.62	6.70E-04	+∞	1,2-dioleoyl-sn-glycero-3-phosphocholine
		760.5875	24.76	3.42	4.70E-04	173.47	PC(16:0/18:1(9Z))
		544.3421	14.72	2.91	4.22E-02	1.72	2-Arachidonoylglycerophosphocholine
		782.5698	29.28	2.73	3.66E-02	8.62	PA(18:2(9Z,12Z)/24:1(15Z))
		808.5838	29.22	2.31	1.35E-03	50.85	PA(24:0/20:4(5Z,8Z,11Z,14Z))
		123.0783	15.44	2.09	1.60E-02	11.54	Nicotinic acid
		149.0233	26.94	1.83	6.28E-03	3.99	1-Methyladenine
		784.5832	29.31	1.63	1.86E-02	3.82	PA(24:1(15Z)/18:1(9Z))
		810.6021	29.27	1.63	3.76E-03	+∞	PA(22:2(13Z,16Z)/22:1(13Z))
		56.0530	27.37	1.57	3.84E-02	1.35	Acrolein
		78.9985	31.59	1.11	7.01E-03	1.21	Pyridine
		194.9650	29.98	1.02	5.12E-03	1.98	Platinum
2-3W↓	Urine	176.0210	14.55	1.37	8.43E-13	0.57	N4-Acetylaminobutanal
		133.0607	3.09	1.36	1.32E-10	0.22	N-Methyl-4-pyridone-3-carboxamide(4PY)
		286.1392	16.48	1.34	3.42E-10	0.51	Urea
		287.1417	16.47	1.33	1.13E-10	0.54	Glycylprolylhydroxyproline
		359.1914	15.84	1.32	2.17E-10	0.37	Fructoselysine
		251.0367	14.04	1.29	1.32E-09	0.56	2,8-Dihydroxyquinoline-beta-D-glucuronide
		151.1436	4.37	1.25	3.02E-08	0.43	Creatinine
		238.0927	11.98	1.17	1.03E-06	0.40	Biopterin
		323.0736	15.05	1.16	2.10E-06	0.51	Indoleacetaldehyde
		222.0965	13.37	1.12	3.55E-08	0.54	N2-Acetyl-L-aminoadipate
		129.0656	14.92	1.23	4.21E-08	0.75	2'-O-Methyladenosine
		267.1190	14.26	1.21	3.55E-07	0.64	Selenocysteine
		214.1297	2.03	1.10	1.01E-05	0.72	Isovalerylsarcosine
	Plasma	303.2261	17.68	1.84	7.71E-08	0.84	Linoleic acid
		414.2581	23.44	1.83	7.71E-06	0.68	3α,12α-Dihydroxy-5β-chol-6-en-24-oic Acid
		381.2965	19.70	1.80	4.53E-09	0.57	MG(0:0/18:0/0:0)
		379.2774	17.43	1.78	7.48E-05	0.80	MG(18:1(9Z)/0:0/0:0)
		866.5846	13.84	1.75	9.21E-03	0.91	LysoPE(0:0/18:0)
		351.2245	17.34	1.67	1.56E-06	0.88	MG(0:0/18:1(11Z)/0:0)
		684.5405	16.76	1.66	4.51E-03	0.49	MG(0:0/16:0/0:0)
		80.9482	0.47	1.74	1.19E-04	0.55	D-Tagatose
	Serum	113.9639	29.33	1.29	4.67E-03	0.62	Hydroxidodioxidosulfidosulfate
		128.9509	29.33	1.10	4.41E-03	0.57	Selenite

VIP: variables importance in the projection values; FC: fold change; Rt: retention time; m/z: mass-to-charge ratio;

**Table 2:** Top 50 differential genes from the comparison of Sham versus MI mice

Gene_name	log2FoldChange	pvalue	padj
Col6a5	-5.106587534	3.55E-29	6.26E-25
Cpz	-3.483426785	2.64E-13	2.33E-09
Cthrc1	-5.34211155	3.10E-11	1.82E-07
Trem2	-2.156973613	1.74E-10	7.69E-07
Anxa8	-3.887173854	3.59E-10	1.27E-06
KMO	-5.094372156	3.71E-10	3.35E-06
Timp1	-3.094041602	6.84E-09	2.01E-05
Fam46b	-1.784008184	2.35E-08	5.91E-05
Aebp1	-2.524560266	3.42E-08	7.53E-05
Tpsb2	-3.54076012	4.18E-08	8.18E-05
Pou3f1	-3.204860229	5.98E-08	0.000105357
Slit2	-1.071752038	8.87E-08	0.000142211
Ildr2	-2.637516087	1.04E-07	0.000152154
Muc16	-3.771936259	1.28E-07	0.000173951
Adamts16	-6.895028006	1.70E-07	0.000214091
Fam180a	-3.278666239	1.83E-07	0.000214356
Gpnmb	-3.340546	1.96E-07	0.000214356
Ephb2	-2.344274809	2.07E-07	0.000214356
Igfbp6	-1.795467822	3.09E-07	0.000303038
Lox	-2.859616809	4.09E-07	0.000367232
Cma1	-3.020266678	4.52E-07	0.000379345
Stac2	-3.097904733	5.88E-07	0.000437574
AC131720.3	3.274126978	6.14E-07	0.000437574
Gpr176	-3.52628993	6.20E-07	0.000437574
Bnc2	-2.431069226	6.21E-07	0.000437574
Hoxc4	-7.014916091	7.06E-07	0.000478496
Marco	-6.929200672	8.24E-07	0.000538217
Igkv14-126	-7.263531139	8.79E-07	0.000553553
P4ha3	-3.846042675	1.06E-06	0.000646957
Flrt2	-1.362220675	1.31E-06	0.000725623
Ighg2c	-6.167310901	1.32E-06	0.000725623
Cemip	-3.915662582	1.32E-06	0.000725623
Il33	-1.94796866	1.64E-06	0.000878322
Gm29216	1.699469385	2.02E-06	0.001047794
Adra2a	-3.57009819	2.29E-06	0.001104086
Steap2	-2.009127292	2.32E-06	0.001104086
Nfasc	-3.167991854	2.32E-06	0.001104086
Postn	-2.92357388	2.53E-06	0.001147203
Krt8	-2.464194121	2.54E-06	0.001147203
Wnt4	-3.777285394	2.66E-06	0.001170355
Grem2	-4.589361908	2.90E-06	0.001246291
Lgi2	-2.238131931	3.13E-06	0.001302165
Itgbl1	-1.907181088	3.22E-06	0.001302165
Nox4	-2.759132495	3.28E-06	0.001302165
Myof	-1.733420986	3.32E-06	0.001302165
Clec4d	-3.548458422	3.45E-06	0.00132027
Bnc1	-4.101519638	3.71E-06	0.001389835
Lrrn4	-3.899149928	3.98E-06	0.001460898
Saa3	-7.135474939	4.67E-06	0.00164523
Myrf	-2.301255295	4.81E-06	0.00164523

**Table 3:** The docking results between compounds with KMO

Protein-ligand	AutoDock score	Hydrogen-bond interaction	Hydrophobic interaction
Ginsenoside Rb3	-13.0 kcal/ mol	Gly16 Gly17 Leu18 Val19 Gly20 Arg40 Ser53 Arg111 Asn115 Gly168 Tyr194 Glu196 Thr198 Thr134 Asp276 Phe278 Leu279 Leu280 Pro281 Ala282 Gln283	Arg40 Ser53 Gln283
Astragaloside IV	-11.0 kcal/ mol	Leu18 Val19 Ser53 Arg111 Gly168 Asp304	Ala39 leu136 Asp167 Ala169 Tyr170
Salvianic acid C	-10.1 kcal/ mol	Arg61 Ser287 His307 Val310 GLU322 Arg349	Phe293 His307 Ile309 Phe313 Gln315 Ala319
Ginsenoside Rg3	-9.2 kcal/ mol	Ala39 Arg135 Asp167 Thr172 Asp304 Ile309 Gly314 Gln315 Gly316	Ile14 Asp167 Ala169 Tyr170
Cryptotanshinone	-9.0 kcal/ mol	Glu322 Arg349	His307 Ile309 Asn318 Asp353
Tanshinone IIA	-8.9 kcal/ mol	Glu322 Arg349	His307 Ile309 Ala319
Emodin	-8.9 kcal/ mol	Ser59 ARG61 Gln315 Glu322 Arg349	Ile309 Gln315 Ala319
Tanshinol A	-8.7 kcal/ mol	Arg61 Phe313 Gln315 Glu322 Arg349	His307 Ile309 Ala319
Salvianic acid D	-8.7 kcal/ mol	Ile286 Ile309 Gly316 Met317	Ala57 Tyr194 Pro311
Hydroxytanshinone IIA	-8.6 kcal/ mol	Ile309 Gly316	Leu56 Ile224 Pro311
Notoginsenoside R1	-8.5 kcal/ mol	Leu136 Asp167 Thr172 Tyr194 Gln283 Pro284 Ile286 Ile309	Phe295
Apigenin	-8.4 kcal/ mol	Ser287 His307 Arg349	Phe293 Ile309
Calycosin	-8.3 kcal/ mol	Ser357 Asp358	Ile224 Leu226 Asp358 Leu359
Formononetin	-8.3 kcal/ mol	Thr236 Ser357 Asp358	Leu226 Pro311 Asp358 Leu359
Luteolin	-8.3 kcal/ mol	Ser53 Pro284 Ile286	Ala169 Tyr194 Thr136 Phe238 Ile286
Ginsenoside Rb1	-8.2 kcal/ mol	Gly17 Leu18 Val19 Gly20 Arg40 Thr43 Ser53 Arg111 Glu112 Ala169	Tyr194 Leu280 Gln283
Rosmarinic acid	-8.2 kcal/ mol	Ser53 Ala57 Ile286 Asp304 Ile309 Met317 Asn318	Met285 Pro311
Neotanshinone A	-7.9 kcal/ mol	Arg61 Glu322 Arg349	Ile309 Gln315 Asn318 Ala319
Tanshinol B	-7.4 kcal/ mol	Ser59 Gln315 Ser357	Gln104 Ile106 Gln315 Ala319
Salvianic acid A	-6.5 kcal/ mol	Tyr37 Ala39 Leu136 Asp167 Tyr170 Thr172	Leu136 Asp167
Caffeic acid	-6.4 kcal/ mol	Ser53 Asp276 Leu279 Leu280 Pro281	Tyr194 Glu196 Leu279 Leu280
Protocatechuic acid	-6.4 kcal/ mol	Tyr37 Glu38 Ala39 Leu136 Tyr170 Thr172	Ile14 Leu136 Asp167
3,4-Dihydroxyphenyl lactic acid (DLA)	-6.1 kcal/ mol	Tyr37 Ala39 Leu136 Asp167 Thr172	Ala39 Arg135 Leu136 Asp167
Protocatechualdehyde	-5.8 kcal/ mol	Glu38 Ala39 His134 Leu136 Thr172	Ile14 Leu136 Asp167

## Abbreviations

MI: myocardial ischemia
CAL: coronary artery ligation
HPLC-Q/TOF-MS: high-performance liquid chromatography and quadrupole time-of-flight mass spectrometry
KAT1: kynurenine aminotransferase 1
KMO: kynurenine 3-monooxygenase
CVD: cardiovascular disease
XA: xanthurenic acid
SV: stroke volume
IVSd: interventricular septum in diastole
LVIDd: left ventricle interior diameter in diastole
LVPWd: left ventricle posterior wall in diastole
LV Vold: left ventricular volume in diastole
LVPEP: left ventricular pre-ejection period
LVET: left ventricular ejection time
EF: ejection fraction
FS: fractional shortening
LV Mass: left ventricular mass
PEP: pre-ejection periodquality
QC: control samples
TICs: total ion chromatograms
OPLS-DA: orthogonal partial least-squares discriminant analysis
MRM: multiple-reaction-monitoring
pAAV9: Serotype 9 adeno-associated virus
NC: negative control
Mfn2: mitofusin-2
OPA1: Optic Atrophy 1
Drp1: Dynamin-relatedprotein1
DAPI: 4':6-Diamidino-2-phenylindole
FHC: Ferritin heavy chain
FLC: Ferritin light chain
GPX4: Glutathione peroxidase
Pink1: PTEN induced putative kinase 1
AR: aspect ratio
FF: form factor
DMEM: Dulbecco's modified eagle medium
FBS: fetal bovine serum
OGD: oxygen and glucose deprivation
SPR: Surface Plasmon Resonance
ROC: receiver operating characteristic
AUC: area under curve
BNP: B type natriuretic peptide
hs-CRP: hypersensitive C-reactive protein
MDA: Malondialdehyde
TNF-α: tumor necrosis factor alpha
LDH: lactate dehydrogenase
NO: nitric oxide
TCA: tricarboxylic acid cycling
AD: Alzheimer's disease
HD: Huntington's disease
OMM: outer mitochondrial membrane
ROS: reactive oxygen species
