# Supplementary material for: Inhibition of KMO Ameliorates Myocardial Ischemia Injury via Maintaining Mitochondrial Fusion and Fission Balance
Source: Int J Biol Sci. 2023 Jun 12;19(10):3077–98. doi: 10.7150/ijbs.83392 (PMC10321280; doi:10.7150/ijbs.83392)
Supplement: Supplementary file 1 — Supplementary figures and tables. [file ijbsv19p3077s1.pdf]

Supplementary Figures

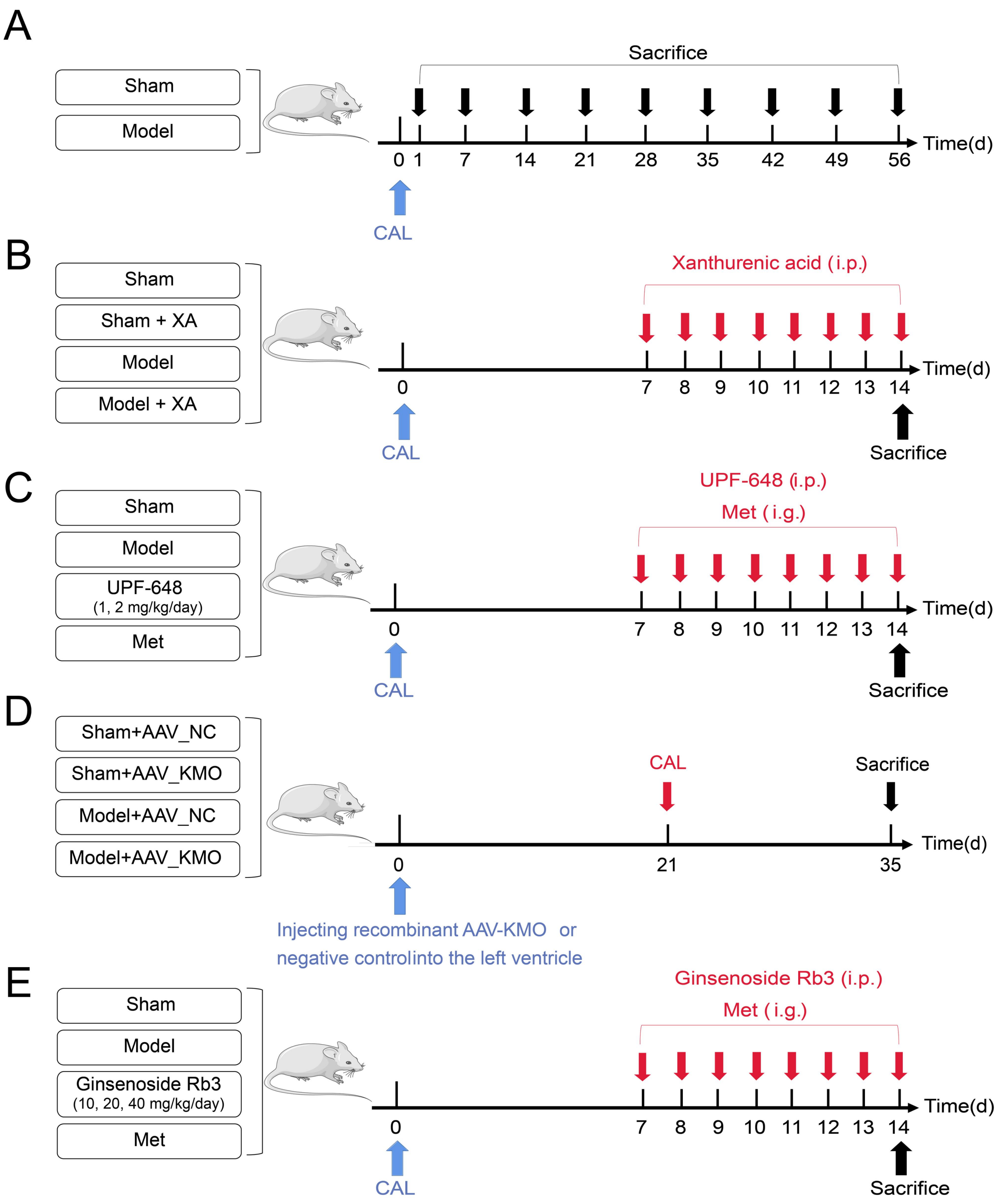

Supplementary Figure 1.

A. The mice were subjected to the MI model by the left anterior descending coronary artery ligation

(CAL) and sacrificed at 1 d, 7 d, 14 d, 21 d, 28 d, 35 d, 42 d, 49 d, and 56 d after CAL.

B&C. The mice were subjected to the MI model by CAL and administrated with (**B**) xanthurenic acid (100 mg/kg, *i.p.*), (**C**) UPF-648 (the inhibitor of KMO) (1 mg/kg/day, 2 mg/kg/day, *i.p.*), and Met (positive control drug) (5.14 mg/kg/day, *i.g.*) on the 7th day after CAL for 8 consecutive days.

D. Adeno-associated virus-packed scrambled (NC) or KMO were injected into the left ventricle of the mice. 21 days later, the mice were subjected to the MI model by CAL for 14 days.

E. Ginsenoside Rb3 (10 mg/kg/day, 20 mg/kg/day, 40 mg/kg/day, *i.g.*), and Met (positive control drug) (5.14 mg/kg/day, *i.g.*) on the 7th day after CAL for 8 consecutive days.

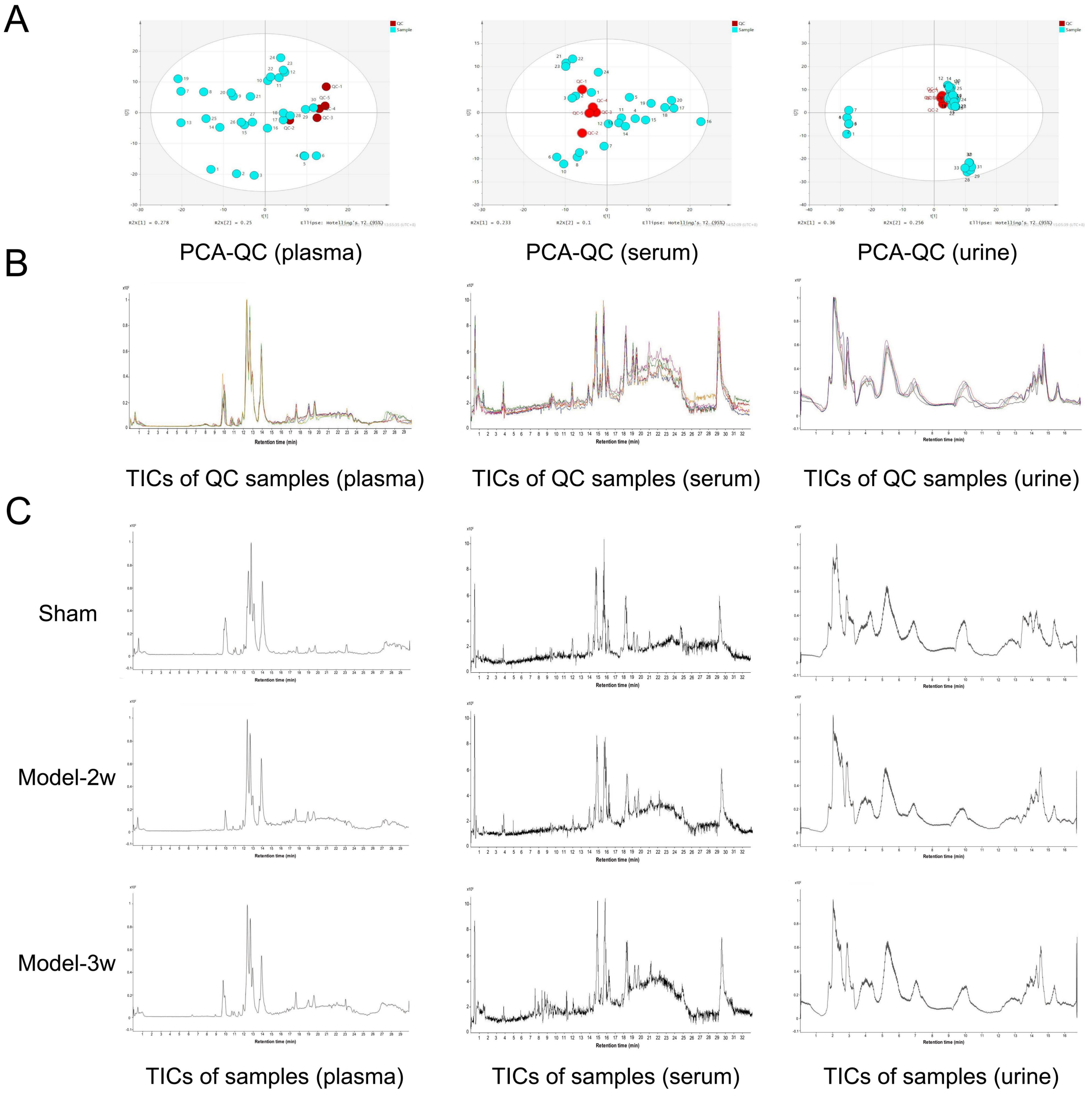

Supplementary Figure 2.

A. Unsupervised PCA analysis of test samples and QC samples of plasma, serum, and urine. The samples were collected on the 14th day after CAL.

B. TICs of QC samples of plasma, serum, and urine.

C. TICs of plasma, serum, and urine samples in sham, model-2w, and model-3w groups.

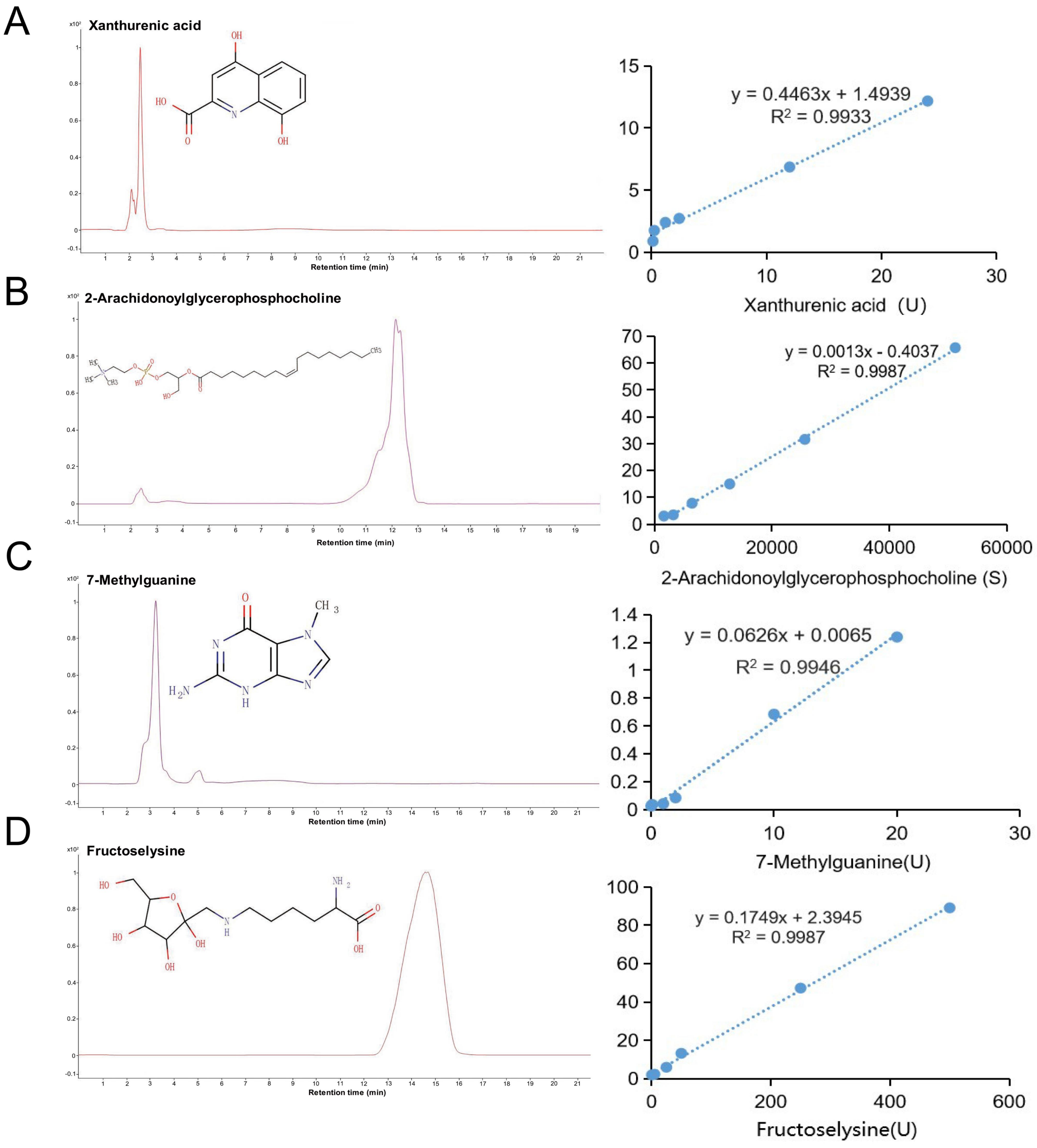

Supplementary Figure 3.

A. Xanthurenic acid.

B. 2-Arachidonoylglycerophosphocholine.

C. 7-Methylguanine.

D. Fructoselysine. All R^2 values were greater than 0.99, which means the linear curve fitted well.

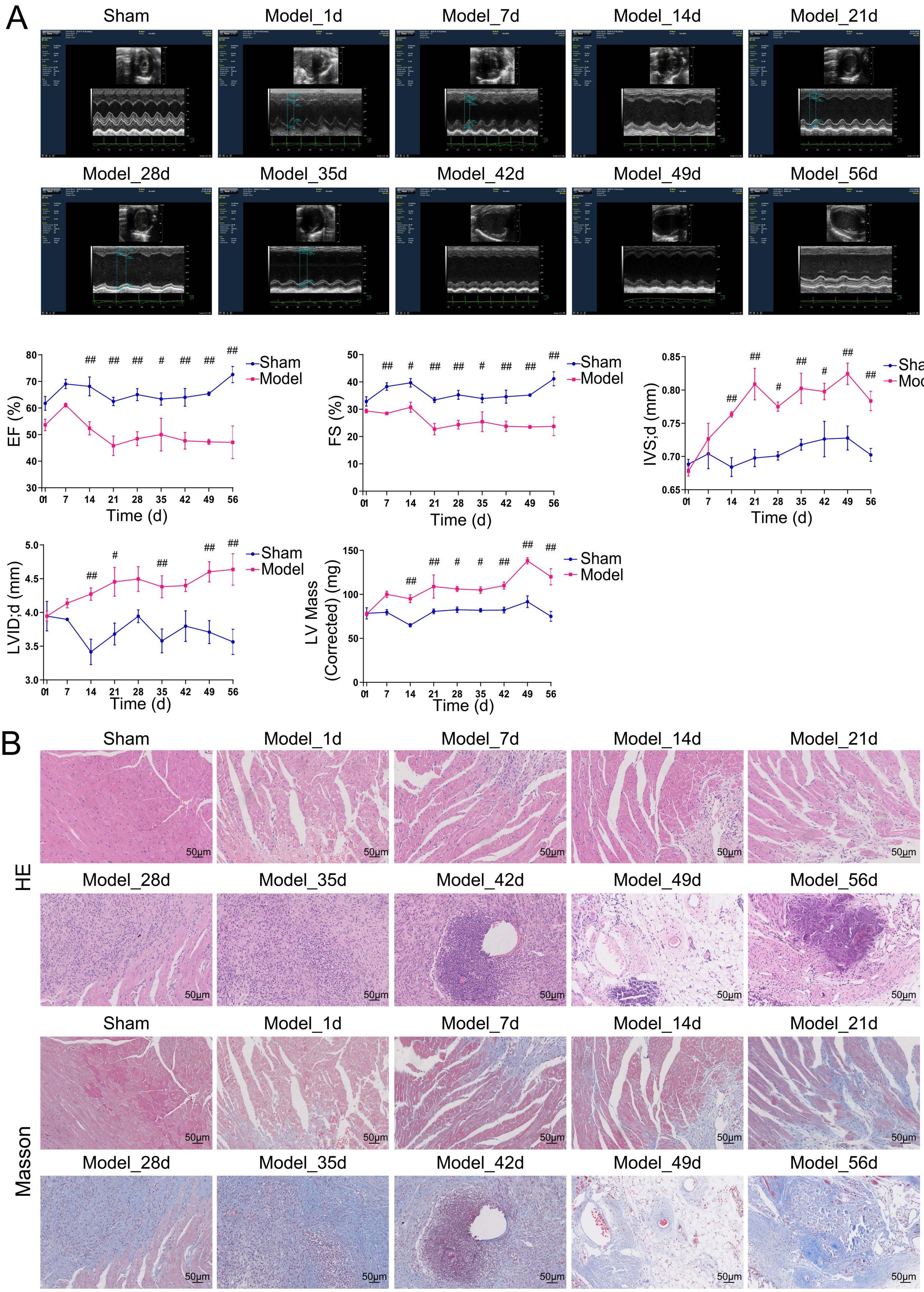

Supplementary Figure 4.

A. M-mode echocardiography and the indicators of cardiac functions in sham and MI model groups mice during CAL 8 weeks. The echocardiographic parameters were measured, including ejection fraction (EF), fractional shortening (FS), interventricular septum (IVS) at diastolic end-stage, left ventricular internal diameter (LVID), and left ventricular mass corrected (n = 6).

B. Changes of myocardial pathology and fibrosis in sham and MI model groups mice during CAL 8 weeks (n = 5) (200X magnification, the lines marked in all figures represent 50 μm). Changes in myocardial pathology were evaluated by HE staining. Changes in myocardial fibrosis were evaluated by Masson staining. Data are presented as mean ± SD. $^{\#}p < 0.05$, $^{\#\#}p < 0.01$ vs. sham group.

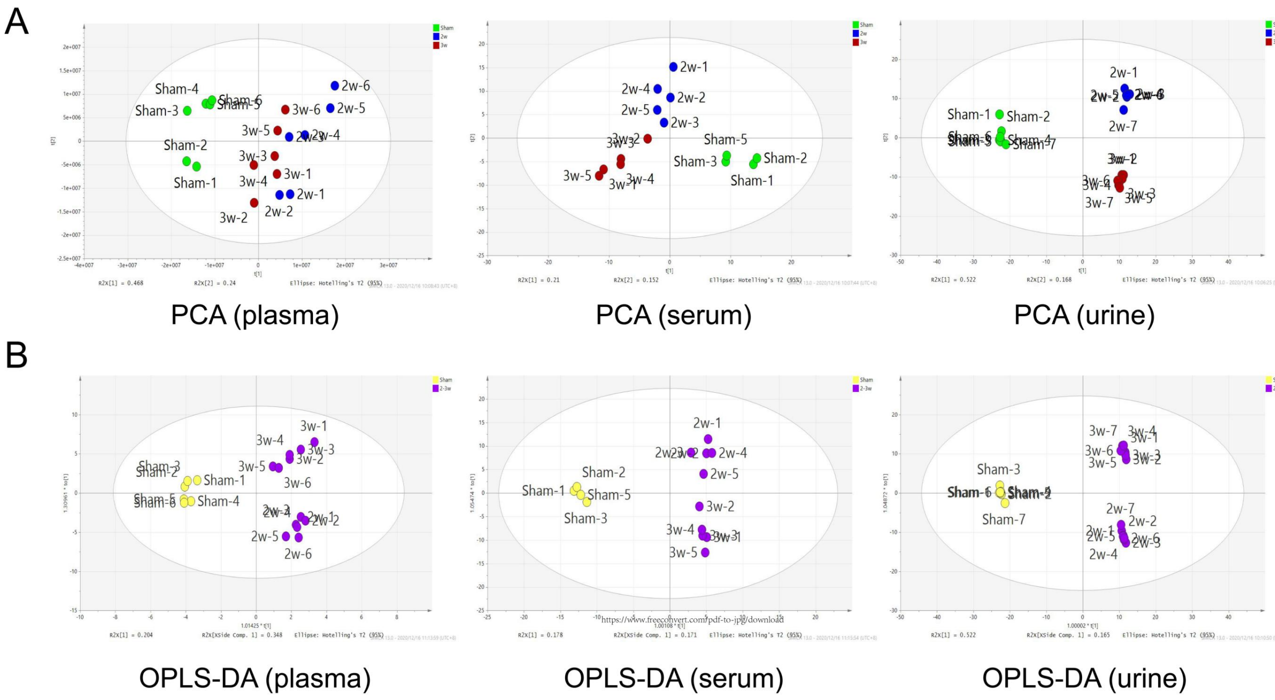

Supplementary Figure 5.

A&B. PCA (**A**) and OPLS-DA (**B**) scores of serum, plasma, and urine samples in sham, MI-2w, and MI-3w groups.

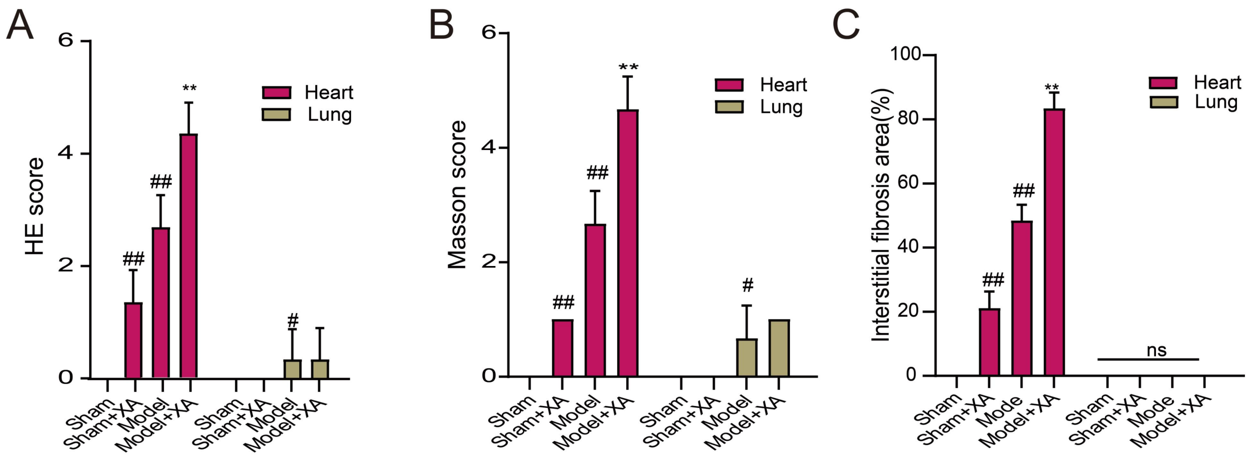

Supplementary Figure 6.

A – C. Quantitative results histological analysis of heart, lung, liver, spleen, kidney, and brain slices by hematoxylin and eosin (H&E) (**A**), masson's trichrome (**B**), and sirius red staining (**C**) (n = 5). Data are presented as mean ± SD. $^{\#}p < 0.05$, $^{\#\#}p < 0.01$ vs. sham group, $^{*}p < 0.05$, $^{**}p < 0.01$ vs. model group; ns means no significance.

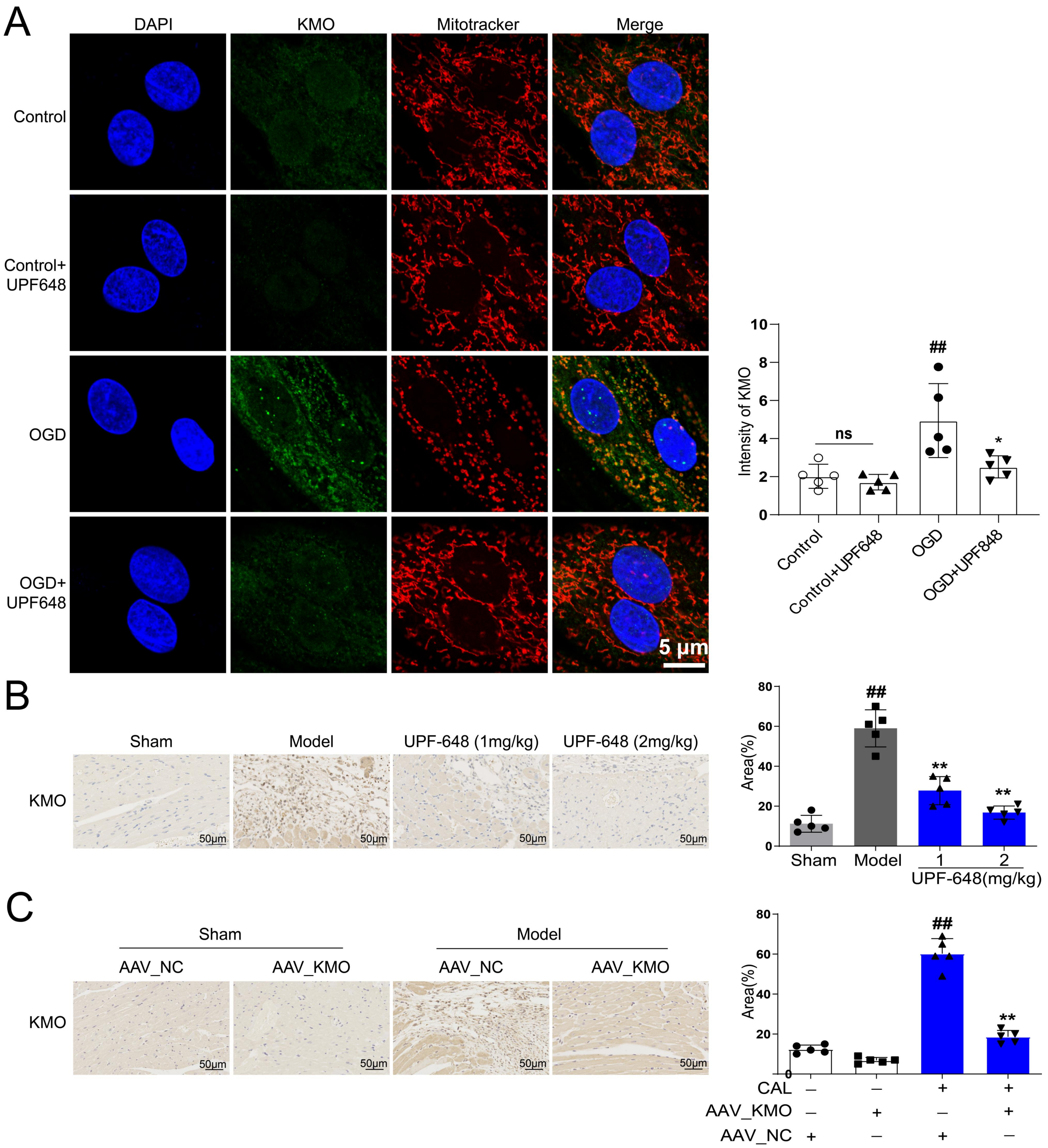

Supplementary Figure 7.

A. Representative immunofluorescence images and statistical results of KMO in H9c2 cells administrated with UPF-648 (2 μmol/L) (n = 5).

B&C. Representative immunohistochemical staining images and statistical results of KMO expression in heart tissue of MI mice treated with KMO inhibitor UPF-648 (1 mg/kg/day and 2

mg/kg/day) and AAV-KMO (400X magnification, the lines marked in all figures represent 50 μm) (n = 5). Data are presented as mean ± SD. $^{\#}p < 0.05$, $^{\#\#}p < 0.01$ vs. control or sham or sham + AAV_NC group, $^{*}p < 0.05$, $^{**}p < 0.01$ vs. OGD or model or model + AAV_NC group; ns means no significance.

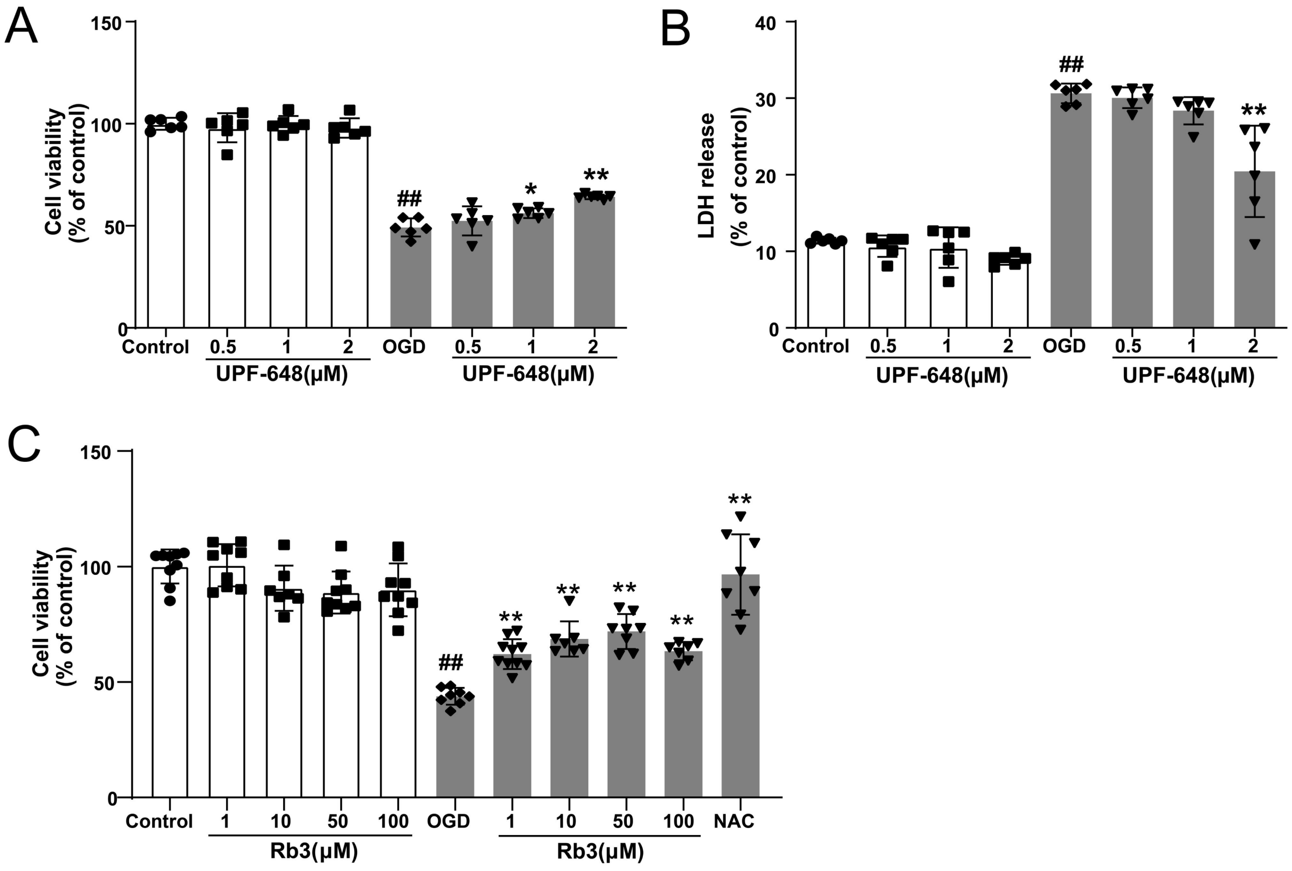

Supplementary Figure 8.

A&B. Protective effects of KMO inhibitor UPF-648 on the viability and up-regulation of LDH release induced by OGD on H9c2 cells (n = 6) (0.5 μM, 1 μM, 2 μM).

C. Protective effect of ginsenoside Rb3 on the cell viability was measured by MTT (n = 7-10) (1 μM, 10 μM, 50 μM, 100 μM). Data are presented as mean ± SD. $^{\#}p<0.05$, $^{\#\#}p<0.01$ vs. control group. $^{*}p < 0.05$, $^{**}p < 0.01$ vs. OGD group.

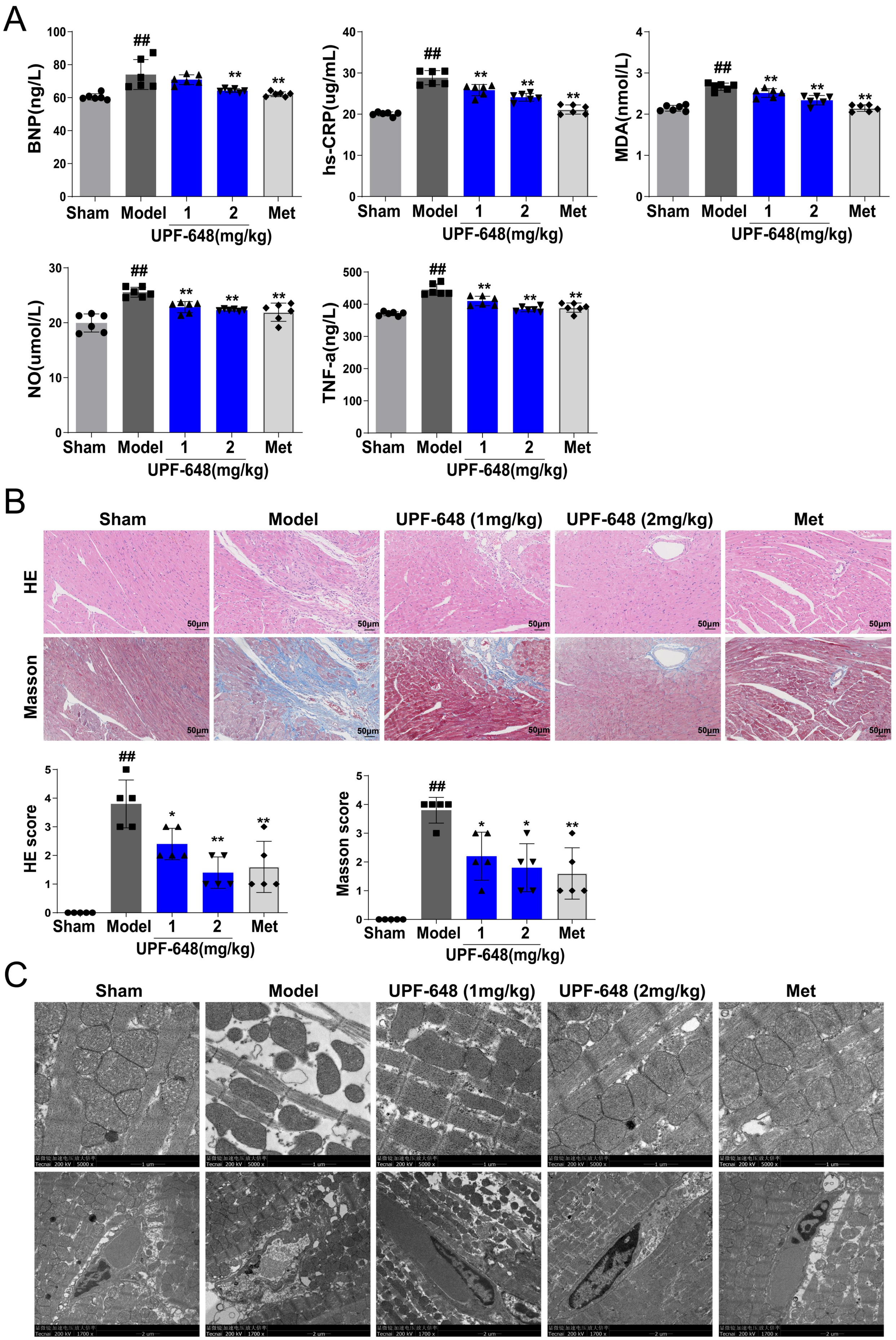
A
BNP(ng/L)
hs-CRP(ug/mL)
MDA(nmol/L)
NO(umol/L)
TNF-a(ng/L)
##
**
Sham
Model
1
2
Met
UPF-648(mg/kg)
B
Sham
Model
UPF-648 (1mg/kg)
UPF-648 (2mg/kg)
Met
HE
Masson
50μm
HE score
Masson score
*
C

Supplementary Figure 9.

A. Serum biochemical indicators contents include BNP, hs-CRP, TNF-α, MDA, and NO in MI mice treated with UPF-648, and were detected by ELISA (n = 6).

B. Changes of myocardial histological features in MI mice treated with UPF-648 (200X magnification, the lines marked in all figures represent 50 μm). Myocardial pathology was evaluated by HE staining (n = 5). Myocardial fibrosis was evaluated by Masson staining (n = 5).

C. Changes of heart ultrastructural in MI mice treated with KMO inhibitor UPF-648, and detected by transmission electron microscopy (n = 3). Data are presented as mean ± SD. $^{\#}p < 0.05$, $^{\#\#}p < 0.01$ vs. sham group. $^{*}p < 0.05$, $^{**}p < 0.01$ vs. model group.

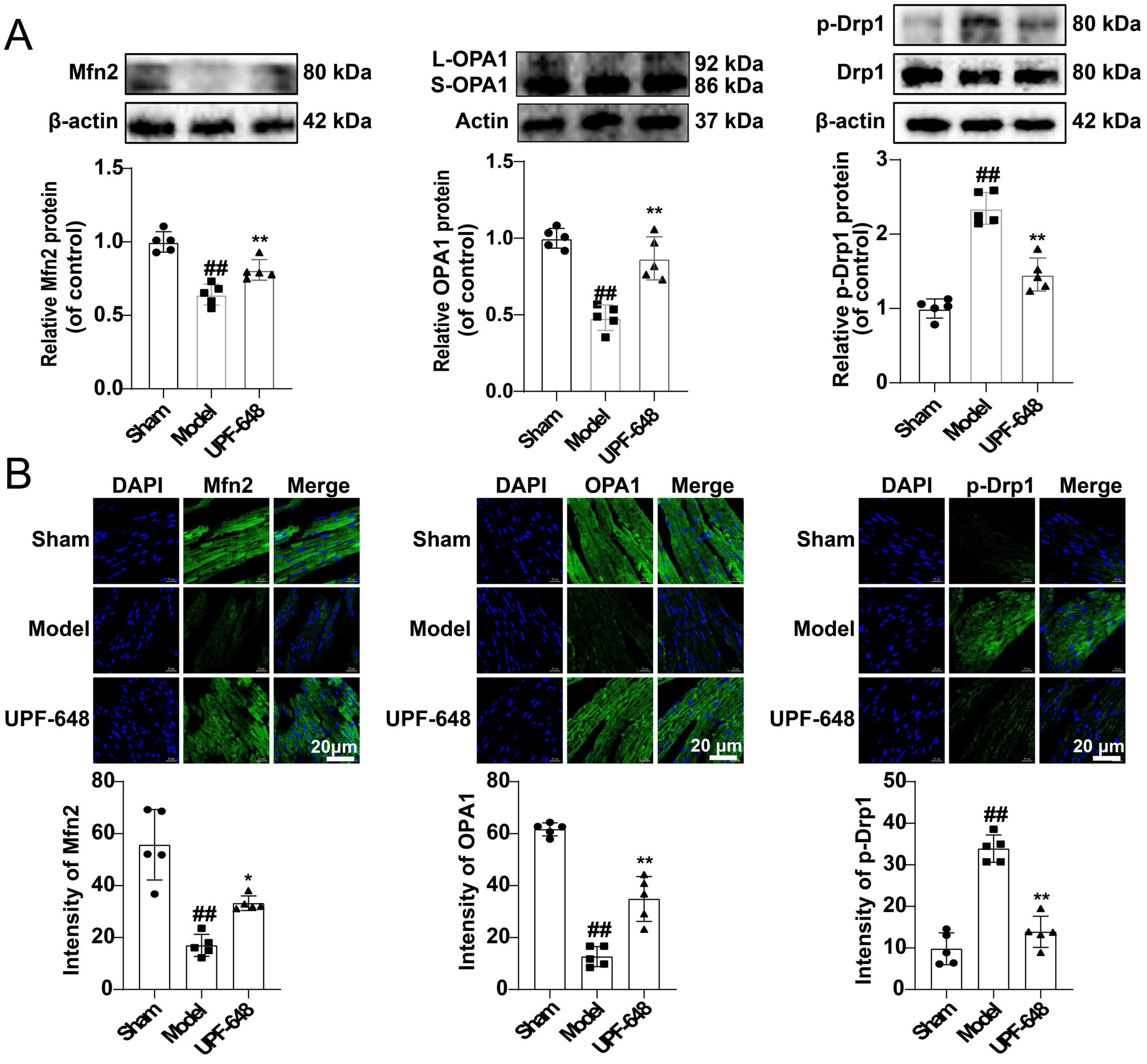

Supplementary Figure 10.

A. The protein expression and relative quantitative data of Mfn2, OPA1, p-Drp1, and Drp1 in heart tissues of MI mice treated with UPF-648, and detected by western blot (2 mg/kg/day) (n = 5).

B. Representative immunofluorescence images and statistical results of Mfn2, OPA1, and p-Drp1 in heart tissues of MI mice treated with UPF-648 (2 mg/kg/day) (n = 5). Data are presented as mean ± SD. $^{\#}p < 0.05$, $^{\#\#}p < 0.01$ vs. sham group, $^{*}p < 0.05$, $^{**}p < 0.01$ vs. model group.

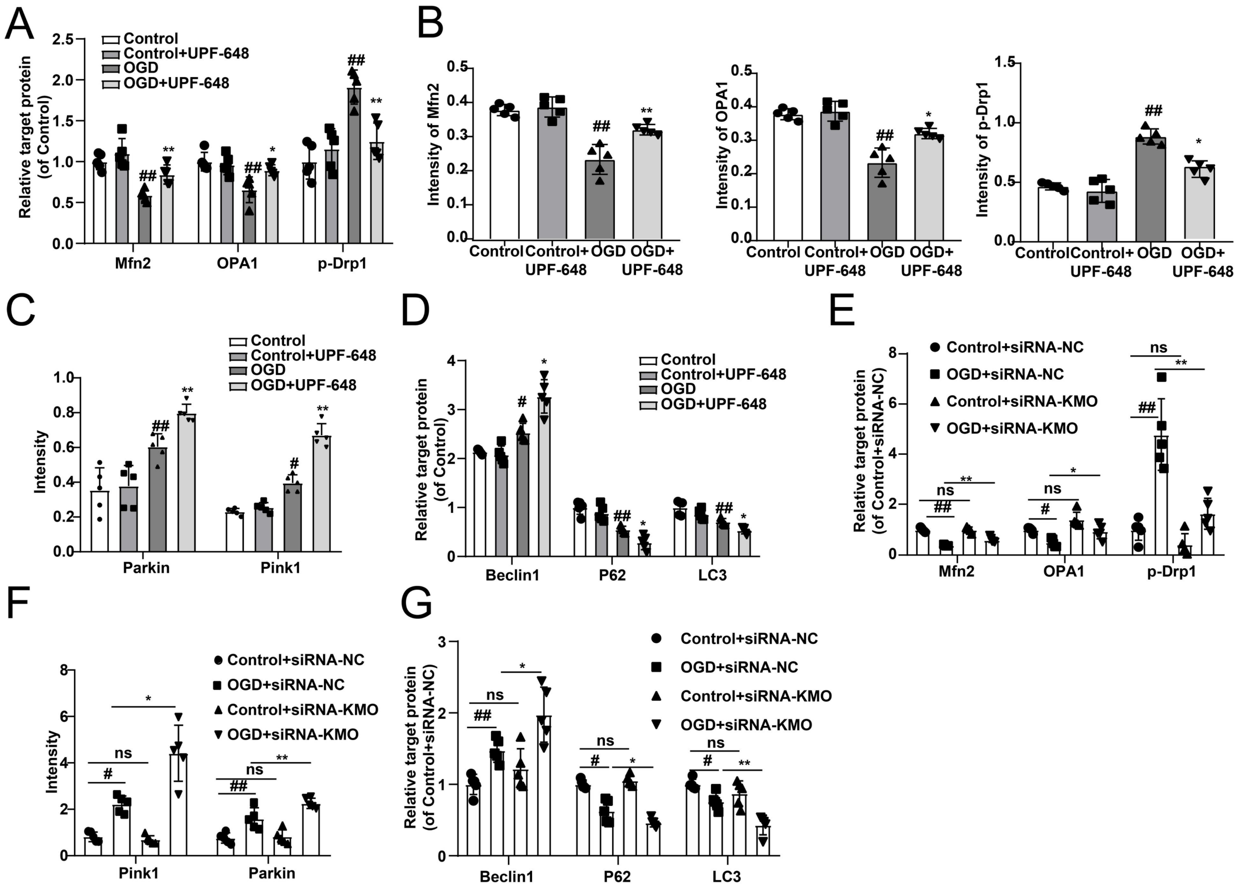

Supplementary Figure 11.

A. Western blotting statistical results of Mfn2, OPA1, and p-Drp1 in OGD-induced H9c2 cells injury model treated with KMO inhibitor UPF648 (2 μmol/L) (n = 5).

B. Immunofluorescence statistical results of Mfn2, OPA1, and p-Drp1 in OGD-induced H9c2 cells injury model treated with KMO inhibitor UPF648 (2 μmol/L) (n = 5).

C. Immunofluorescence statistical results of Pink1 and Parkin in OGD-induced H9c2 cells injury model treated with KMO inhibitor UPF648 (2 μmol/L) (n = 5).

D. Western blotting statistical results of Beclin1, P62, and LC3 I/II in OGD-induced H9c2 cells injury model treated with KMO inhibitor UPF648 (2 μmol/L) (n = 5).

E. Western blotting statistical results of Mfn2, OPA1, and p-Drp1 in OGD-induced H9c2 cells injury model in the OGD-induced H9c2 cells injury model treated with siRNA-KMO or siRNA-NC

(n = 5).

F. Immunofluorescence statistical results of Pink1 and Parkin in OGD-induced H9c2 cells injury model treated with siRNA-KMO or siRNA-NC (n = 5).

G. Western blotting statistical results of Beclin1, P62, and LC3 I/II in OGD-induced H9c2 cells injury model treated with siRNA-KMO or siRNA-NC (n = 5). Data are presented as mean ± SD. $^{\#}p < 0.05$, $^{\#\#}p < 0.01$ vs. control group or control + siRNA_NC group, $^{*}p < 0.05$, $^{**}p < 0.01$ vs. OGD group or OGD + siRNA_NC group; ns means no significance.

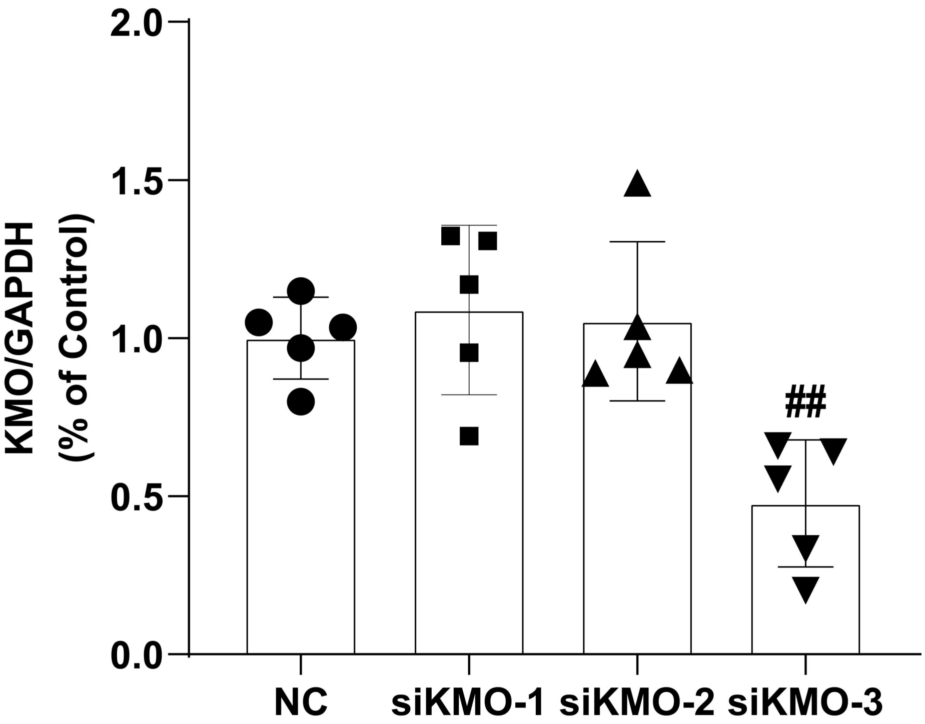

Supplementary Figure 12.

Representative western blotting analysis of the expression of KMO in H9c2 cells transfected with KMO-siRNA (n = 5). Data are presented as mean ± SD. $^{##}p < 0.01$ vs. NC group.

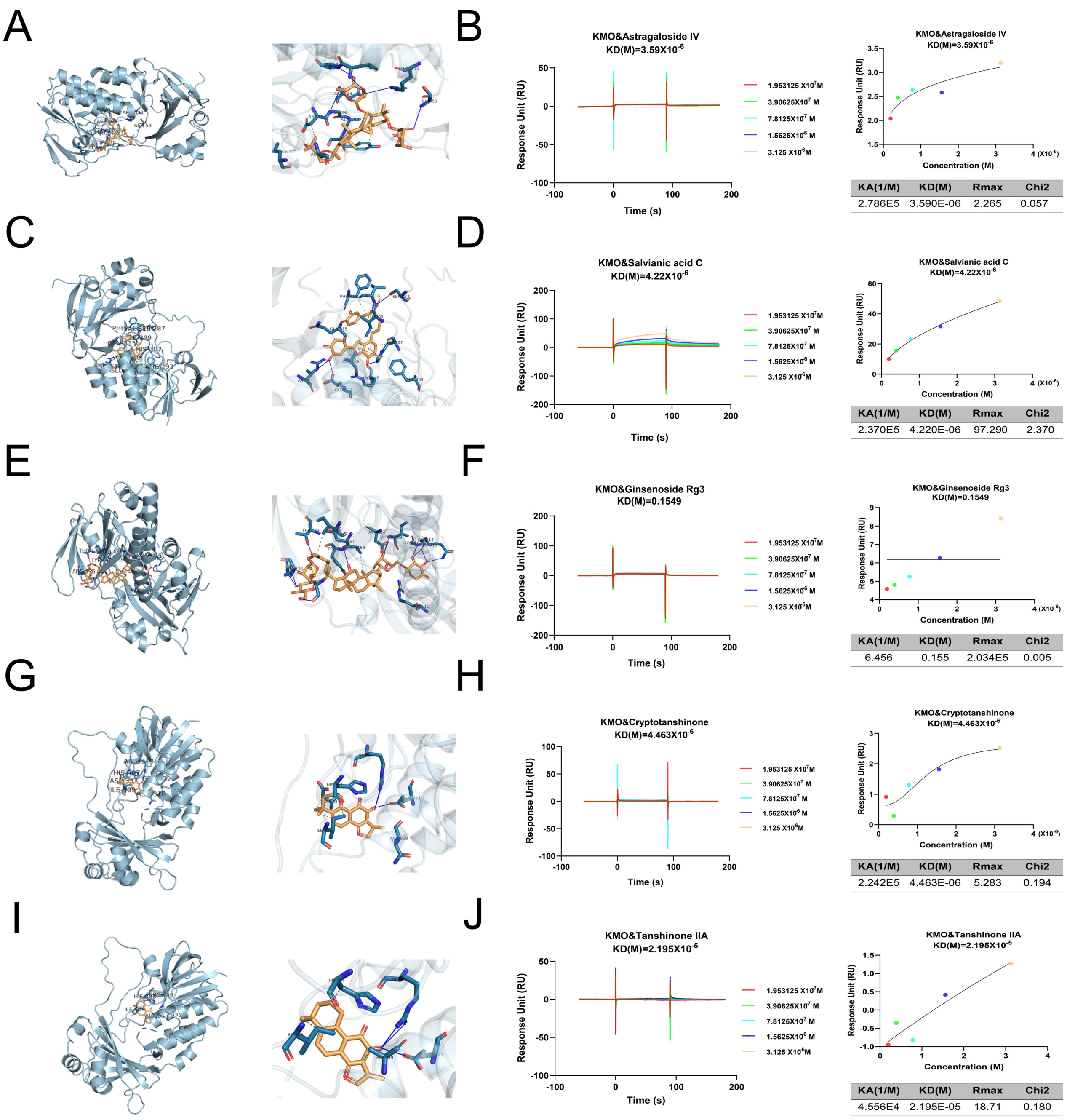

Supplementary Figure 13.

A. Molecular docking model carried out by Autodock vina revealed that Astragaloside IV bonded to the KMO.

B. SPR analysis of the binding affinity of Astragaloside IV and KMO protein. The Kd (mol/L) value between Astragaloside IV and KMO was 3.59×10^{-6} mol/L.

C. Molecular docking model revealed that Salvianic acid C bonded to the KMO.

D. SPR analysis of the binding affinity of Salvianic acid C and KMO protein. The Kd (mol/L) value between Salvianic acid C and KMO was 4.22×10^{-6} mol/L.

E. Molecular docking model revealed that Ginsenoside Rg3 bonded to the KMO.

F. SPR analysis of the binding affinity of Ginsenoside Rg3 and KMO protein. The Kd (mol/L) value between Ginsenoside Rg3 and KMO was 0.1549 mol/L.

G. Molecular docking model c revealed that Cryptotanshinone bonded to the KMO.

H. SPR analysis of the binding affinity of Cryptotanshinone and KMO protein. The Kd (mol/L) value between Cryptotanshinone and KMO was 4.463×10^{-6} mol/L.

I. Molecular docking model revealed that Tanshinone IIA bonded to the KMO.

J. SPR analysis of the binding affinity of Tanshinone IIA and KMO protein. The Kd (mol/L) value between Tanshinone IIA and KMO was 2.195×10^{-5} mol/L.

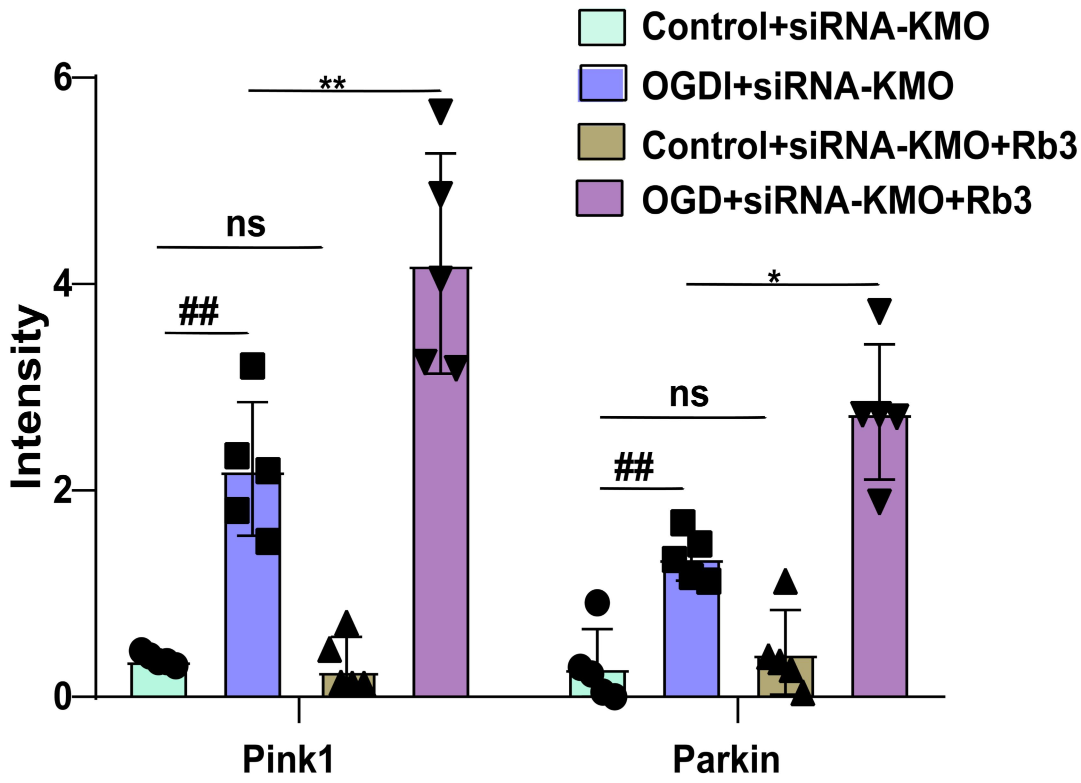

Supplementary Figure 14.

Immunofluorescence statistical results of Pink1 and Parkin in the OGD-induced H9c2 cells injury model treated with siRNA-KMO or ginsenoside Rb3 (n = 5). Data are presented as mean ± SD. $^{\#}p < 0.05$, $^{\#\#}p < 0.01$ vs. control + siRNA-KMO group, $^{*}p < 0.05$, $^{**}p < 0.01$ vs. OGD + siRNA-KMO group; ns means no significance.

Supplementary Tables

Table S1. Clinical characteristics of MI patients and healthy controls

Clinical characteristics	Healthy controls	MI patients	p value
Age, yrs	59.73±4.72	61.34 ±16.09	7.1e-02
Male/female	8/15	19/22	—
Pulse rate, times/min	74.61 ± 3.72	77.18 ± 5.45	5.1e-01
Respiratory rate, times/min	17.03 ± 2.03	17.79 + 1.34	7.3e-01
Systolic blood pressure, mm Hg	117.97 ± 1.45	120.27 ± 0.33	2.1e-01
Diastolic blood pressure, mm Hg	67.98 ± 3.28	71.64 ± 4.83	9.8e-02
LVEF, %	61 ± 2.44	32 ± 2.16	2.4e-03
Glucose, mmol/L	7.43 ± 2.52	7.06 ± 0.41	6.9e-01
Total cholesterol, mmol/L	4.13 ± 0.16	4.67 ± 0.73	7.4e-01
hs-CRP, mg/L	4.96 ± 0.11	12.06 ± 3.01	1.3e-03
CK-MB, U/L	9.57 ± 0.37	22.33 ± 1.98	5.8e-04
LDH, U/L	161.08 ± 9.95	271.18 ± 19.14	7.7e-03
BNP, pg/ml	61.44 ± 3.35	871.39 ± 51.32	2.5e-06

Table S2. The relative standard deviation (RSD%) of retention time and peak area in QC samples

Sample	m/z	RSD of retention time(%)	RSD of Peak area(%)
	130.0070	3.5336	12.7461
	230.2453	0.2858	18.8795
Serum	235.1684	0.2395	8.3588
	237.2215	0.3376	13.0289
	247.2421	0.1728	6.7976
	1245.0385	0.4336	6.0505
	524.3712	0.0193	1.8923
Plasma	991.6721	0.0226	0.4163
	360.3239	0.2027	5.7742
	1047.7350	0.0193	1.3693
	338.3402	0.0646	4.0527
	792.5873	2.2387	6.8497
Urine	114.0658	0.7125	2.6875
	166.0716	1.0018	4.7207
	132.0762	0.1222	3.2929

Table S3. Representative standard curve equatio and linear range of metabolites

Metabolites	Representative standard curve equation	Linear range
Fructoselysine(U)	y = 0.1749x + 2.3945（R^2 =0.9987）	0.5-500 μg/mL
7-Methylguanine(U)	y = 0.0626x + 0.0065（R^2 =0.9946）	0.02-20 μg/mL
Xanthurenic acid（U）	y = 0.4463x + 1.4939（R^2 =0.9933）	0.12-24 μg/mL
2-Arachidonoyl glycero phoshphocholine(S)	y = 0.0013x - 0.4037（R2 = 0.9987）	1600-51200 ng/mL

U: urine; S:serum.

Table S4. Method validation for the targeted analysis of metabolites

Metabolite	Ion mode	Q1	Q3	Fragmentor (v)	CE (v)	LOQ (ng/mL)	LOD (ng/mL)	Extraction recovery (%)	Matrix effect (%)	Precision(RSD%)		Accuracy(%)		Concentration of QC
										Within-day	Between-day	Within-day	Between-day	
Fructoselysine(U)	Positive	309.2	225.3	100	5	5.05	1.52	82.97	115.60	3.96	6.68	7.21	6.73	250μg/mL
7-Methylguanine(U)	Positive	166.1	149.1	100	9	0.06	0.02	99.97	47.40	10.19	24.59	7.94	7.48	2μg/mL
Xanthurenic acid（U）	Positive	206.0	160.0	90	9	0.01	0.00	103.11	192.75	10.10	14.67	3.75	12.25	2.4μg/mL
2-Arachidonoyl glycero phoshphocholine(S)	Positive	545.4	184.2	135	20	0.06	0.02	103.55	12.91	9.80	11.38	2.81	12.08	25.6μg/mL

LOQ: limit of quantitation.

LOD: limit of detection.

CE: collision energy.

U: urine; S:serum.

Table S5. Echocardiographic indicators in sham and MI mice during CAL 8 weeks (n = 6).

	Sham									Model								
**Day**	**1**	**7**	**14**	**21**	**28**	**35**	**42**	**49**	**56**	**1**	**7**	**14**	**21**	**28**	**35**	**42**	**49**	**56**
IVS;d (mm)	0.69±0.02	0.70±0.05	0.68±0.031	0.70±0.03	0.70±0.01	0.72±0.02	0.73±0.06	0.73±0.04	0.70±0.02	0.68±0.02	0.73±0.05	0.76±0.01	0.81±0.05	0.77±0.02	0.80±0.05	0.80±0.03	0.82±0.04	0.78±0.03
LVID;d (mm)	3.95±0.44	3.90±0.03	3.42±0.38	3.68±0.33	3.94±0.19	3.58±0.35	3.80±0.45	3.71±0.34	3.56±0.38	3.95±0.15	4.14±0.14	4.27±0.18	4.45±0.43	4.50±0.37	4.38±0.33	4.40±0.18	4.60±0.30	4.64±0.47
PEP (ms)	13.5±1.81	16.85±1.42	16.37±1.17	17.00±1.12	15.40±0.56	16.50±1.71	15.33±1.12	15.17±1.09	10.83±2.50	9.83±2.53	15.67±0.95	11.67±1.32	16.36±1.56	19.50±2.33	20.83±2.64	20.17±2.79	19.33±1.09	18.50±1.99
LVPW;d（mm）	0.69±0.03	0.71±0.03	0.68±0.02	0.72±0.05	0.73±0.04	0.73±0.01	0.70±0.02	0.74±0.03	0.68±0.03	0.74±0.03	0.91±0.07	0.79±0.02	0.82±0.06	0.88±0.07	0.95±0.08	0.95±0.03	0.91±0.03	0.76±0.045
LV EF (%)	61.78±5.22	69.08±3.53	68.12±7.09	62.47±3.15	65.04±4.60	63.40±4.62	64.03±6.69	65.38±1.38	72.60±6.04	53.66±4.37	61.10±1.40	52.40±4.91	45.82±7.39	48.49±5.30	50.03±12.39	47.69±6.28	47.26±1.94	47.12±12.25
LV FS (%)	32.86±3.45	38.31±2.79	39.68±3.07	33.38±1.96	35.29±3.26	33.94±3.08	34.61±4.78	35.19±0.80	41.15±5.10	29.33±1.48	28.47±0.82	30.71±3.67	22.73±4.16	24.34±3.21	25.43±7.26	23.78±3.74	23.51±1.12	23.74±6.80
SV (μL)	47.28±5.67	46.23±3.47	47.74±4.56	43.39±3.36	49.17±4.18	46.84±2.89	48.20±1.53	44.64±2.95	50.89±6.99	32.99±4.53	37.15±2.66	37.70±7.74	31.85±3.58	41.20±6.81	37.09±2.85	34.31±1.36	35.12±3.94	41.02±3.79
LV Mass (Corrected) (mg)	78.45±12.75	79.64±6.05	64.95±3.88	80.66±5.35	82.61±6.42	81.91±4.14	82.17±6.44	91.72±13.38	75.00±11.46	77.73±4.69	100.08±7.06	95.10±8.98	108.99±26.34	106.18±5.83	104.99±7.37	110.12±8.18	138.15±7.08	120.06±18.35

Table S6. Echocardiographic indicators in MI mice with cardiac-specific knockdown of KMO (n = 6).

	Sham + AAV-NC	Sham + AAV-KMO	Model + AAV-NC	Model + AAV-KMO
IVS;d (mm)	0.82 ±0.10	0.78 ±0.07	0.91±0.10	0.83±0.08
LVID;d (mm)	3.47±0.21	3.49 ± 0.25	3.81±0.20	3.77±0.16
LVPW;d (mm)	0.71±0.09	0.77±0.11	0.87±0.13	0.86±0.08
LV EF (%)	69.01 ± 6.81	71.04±6.29	42.04±2.37	61.48±5.02
LV FS (%)	38.15±5.43	39.79 ±5.12	20.16±1.38	32.50±3.48
SV (μL)	36.15±4.55	38.05±6.53	23.64±2.64	37.45±5.67

Table S7. Echocardiographic indicators in MI mice treated with ginsenoside Rb3 (n = 5-6).

	Sham	Model	Rb3 (10 mg/kg)	Rb3 (20 mg/kg)	Rb3 (40 mg/kg)	Sham+Rb3 (40 mg/kg)	Met
IVS;d (mm)	0.86±0.10	0.95±0.12	0.96±0.11	0.83±0.17	0.89±0.094	0.85±0.16	0.82±0.08
LVID;d (mm)	3.45±0.40	4.37±0.73	4.05±0.46	4.26±0.43	4.03±0.33	3.87±0.30	3.94±0.43
LVPW;d (mm)	0.98±0.17	1.29±0.28	0.91±0.073	0.90±0.126	0.87±0.055	0.92±0.17	0.94±0.16
LV EF (%)	50.60±8.94	31.86±4.05	39.10±3.28	48.89±5.42	50.51±5.68	47.98±3.53	48.82±4.06
LV FS (%)	25.23±5.41	14.98±2.05	18.70±1.69	24.51±3.21	25.44±3.69	23.73±2.13	24.13±2.47
SV (μL)	29.63±6.41	23.37±5.27	28.40±6.55	40.01±9.41	39.34±8.19	31.04±7.35	33.54±4.42
